# A single diiron enzyme catalyses the oxidative rearrangement of tryptophan to indole nitrile

**DOI:** 10.1038/s41557-024-01603-z

**Published:** 2024-09-16

**Authors:** Sanjoy Adak, Naike Ye, Logan A. Calderone, Meng Duan, Wilson Lubeck, Rebecca J. B. Schäfer, April L. Lukowski, K. N. Houk, Maria-Eirini Pandelia, Catherine L. Drennan, Bradley S. Moore

**Affiliations:** 1 Center for Marine Biotechnology and Biomedicine, Scripps Institution of Oceanography, University of California San Diego, La Jolla, CA, USA.; 2 Department of Chemistry, Massachusetts Institute of Technology, Cambridge, MA, USA.; 3 Department of Biochemistry, Brandeis University, Waltham, MA, USA.; 4 Department of Chemistry and Biochemistry, University of California, Los Angeles, CA, USA.; 5 Department of Biology, Massachusetts Institute of Technology, Cambridge, MA, USA.; 6 Howard Hughes Medical Institute, Massachusetts Institute of Technology, Cambridge, MA, USA.; 7 Skaggs School of Pharmacy and Pharmaceutical Sciences, University of California at San Diego, La Jolla, CA, USA.; 8 These authors contributed equally: Sanjoy Adak, Naike Ye.

## Abstract

Nitriles are uncommon in nature and are typically constructed from oximes through the oxidative decarboxylation of amino acid substrates or from the derivatization of carboxylic acids. Here we report a third nitrile biosynthesis strategy featuring the cyanobacterial nitrile synthase AetD. During the biosynthesis of the eagle-killing neurotoxin, aetokthonotoxin, AetD transforms the 2-aminopropionate portion of 5,7-dibromo-l-tryptophan to a nitrile. Employing a combination of structural, biochemical and biophysical techniques, we characterized AetD as a non-haem diiron enzyme that belongs to the emerging haem-oxygenase-like dimetal oxidase superfamily. High-resolution crystal structures of AetD together with the identification of catalytically relevant products provide mechanistic insights into how AetD affords this unique transformation, which we propose proceeds via an aziridine intermediate. Our work presents a unique template for nitrile biogenesis and portrays a substrate binding and metallocofactor assembly mechanism that may be shared among other haem-oxygenase-like dimetal oxidase enzymes.

The nitrile functional group with its short, polarized C≡N triple bond, is a common feature of medicinal compounds due to its favourable physicochemical and pharmacokinetic properties, metabolic stability, and ability to serve as a bioisostere of carbonyls and halogens^1,2^. Many nitrile-containing pharmaceuticals are approved by the US Food and Drug Administration and used for the treatment of several diseases, including heart failure, hypertension, chronic myeloid leukaemia, breast cancer and fungal infections^1,2^. Given the importance of nitriles in medicinal chemistry, numerous synthetic methods have been established to install nitrile functionality^3^. Nature’s biosynthetic strategies, however, are lesser known because natural nitrile-containing compounds are relatively uncommon and represent approximately 0.1% of natural products. The most common naturally occurring nitriles are cyanogenic glycosides from plants, in which they function as defensive agents^4,5^. Nitriles are also found in arthropods, bacteria and fungi, in which they serve diverse roles as secondary metabolites^6,7^.

The biosynthetic pathways for nitriles are only sparsely described. In plants, *N*-hydroxylation and subsequent decarboxylation of an amino acid precursor gives rise to an aldoxime intermediate that becomes dehydrated to yield the nitrile (that is, cyanogenic glycoside); one such example is the conversion of l-tyrosine (**1**) to (*S*)-dhurrin (**3**) (Fig. 1a). Both aldoxime formation and dehydration are catalysed by haem-containing enzymes, including dedicated cytochrome P450s^8–10^. In bacteria, the ATP-dependent ToyM is a nitrile-forming enzyme that converts a carboxylic acid to a nitrile via an amide intermediate, resulting in the formation of the antibiotic toyocamycin (**6**) (Fig. 1a)^11^. Although there are proposed gene candidates for nitrile formation in the microbial biosynthesis of the enediyne natural product cyanosporaside^12^ and the macrolide borrelidin^13^, the exact reaction pathways have not been yet delineated.

We recently reported a nitrile synthase of cyanobacterial origin, AetD, that converts 5,7-dibromo-l-tryptophan (**7**) to 5,7-dibromo-indole-3-carbonitrile (**8**) during the biosynthesis of aetokthonotoxin^14^ (**9**) (Fig. 1a), a cyanotoxin known for being responsible for the death of eagles^15^. Although we previously showed that the nitrogen atom from the α-amine of **7** is retained in the nitrile product^14^, the origin of the nitrile carbon and the fate of the excised carbon atoms were not established. A single-enzyme-mediated conversion of tryptophan to indole-3-carbonitrile is unprecedented and offers a mechanistic template for coupling nitrile formation with carbon deletion biochemistry. AetD shows no homology to ToyM or to cytochrome P450s, even to those associated with the formation of the structurally similar indole-3-acetonitrile during the biosynthesis of the plant phytoalexin camalexin^16^. AetD instead uses non-haem iron for the conversion of the 2-aminopropionate region of 5,7-dibromo-l-tryptophan to a nitrile.

This study dissects the structural and mechanistic features of the AetD-catalysed reaction via crystallographic analysis of AetD, mechanistic studies employing isotopically labelled substrates, and spectroscopic and kinetic characterization of reaction intermediates. Our work establishes AetD as another member of the haem-oxygenase-like dimetal oxidase (HDO) superfamily^17^, for which chemical transformations include: *N*-oxygenation (SznF^18^, RohS^19^, FlcE^20^), desaturase-lyases (UndA^21^, BesC^22^), methine excision (FlcD^20^) (Fig. 1b), and the conversion of a protein-derived tyrosine side chain to *para*-aminobenzoate as part of the biosynthesis of folic acid in *Chlamydia trachomatis* (CADD)^23^. The HDO enzymes can be classified into two subgroups depending on whether the O_2_-reactive complex forms in the absence or in the presence of substrate (that is, whether it is substrate-independent or substrate-triggered, respectively). In substrate-independent HDOs (for example, SznF)^24,25^, assembly of the diiron cofactor and formation of the O_2_-reactive complex do not require substrate binding. On the other hand, in substrate-triggered HDOs (for example, UndA and BesC)^26–28^, substrate binding probably precedes or facilitates cofactor binding. Here we show that AetD is substrate-triggered and catalyses a reaction that does not match those previously reported for homologous enzymes, thus expanding the functional repertoire of the HDO superfamily.

## Results

### AetD is a member of the HDO structural superfamily

**Extended Data Fig. 1 |:**
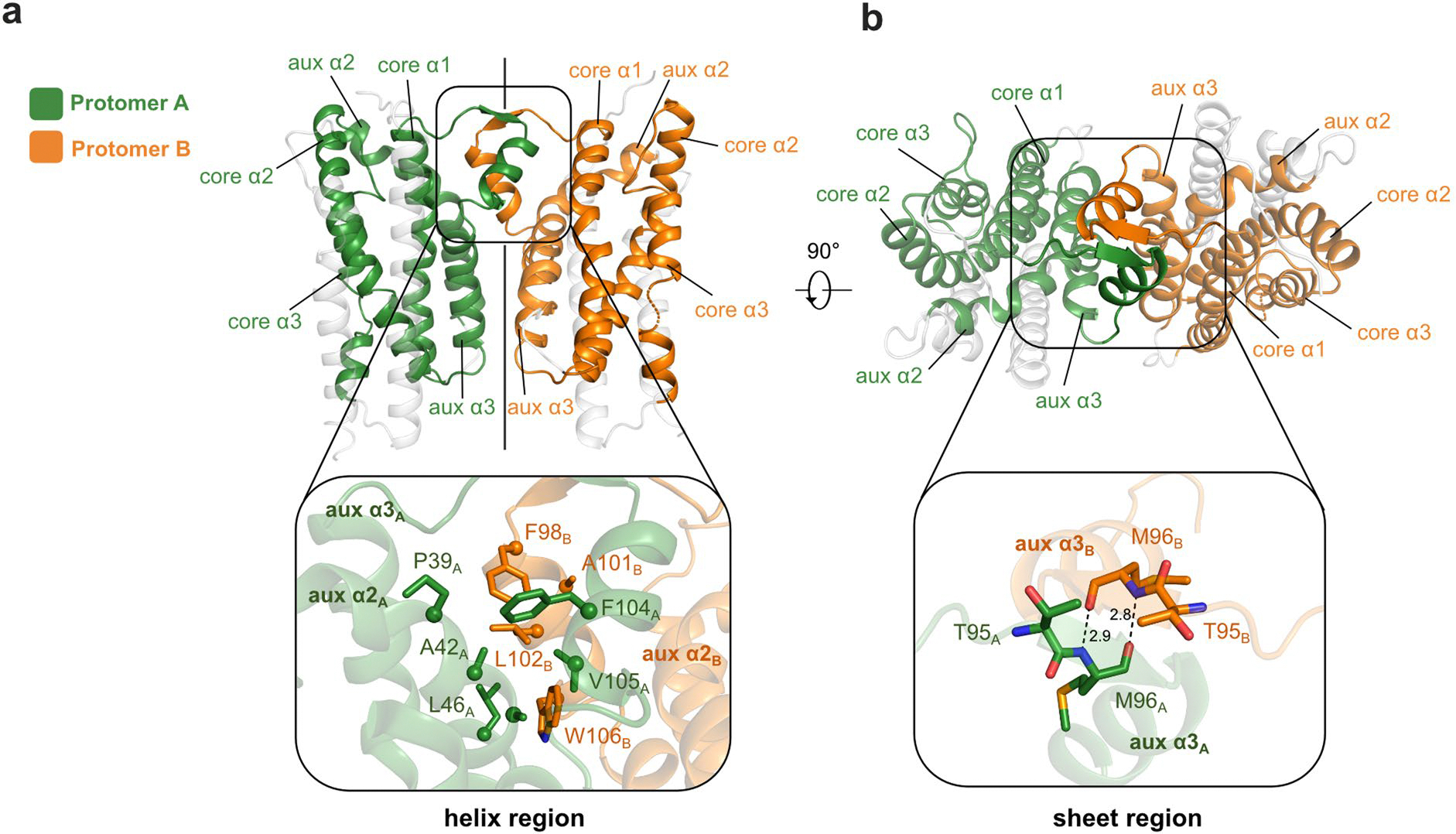
Dimeric structure of AetD and the dimer interface. **a**, (top) The dimeric structure of AetD. Part of the auxiliary helix α3 forms the dimerization domain (enclosed by the black the box) that consists of a helix region and a sheet region. (bottom) zoomed-in view of the helix region. Only half of this 2-fold symmetrical dimer interface is shown for simplicity. The helix region is largely hydrophobic, containing hydrophobic residues from the dimerization domain and auxiliary helix α2 from both protomers. **b**, the dimeric structure rotated 90° out of plane highlighting the sheet region of the dimerization domain. (bottom) zoomed-in view of the sheet region, which is made of two residues from each protomer (T95 and M96) held together by backbone hydrogen bonds.

**Extended Data Fig. 2 |:**
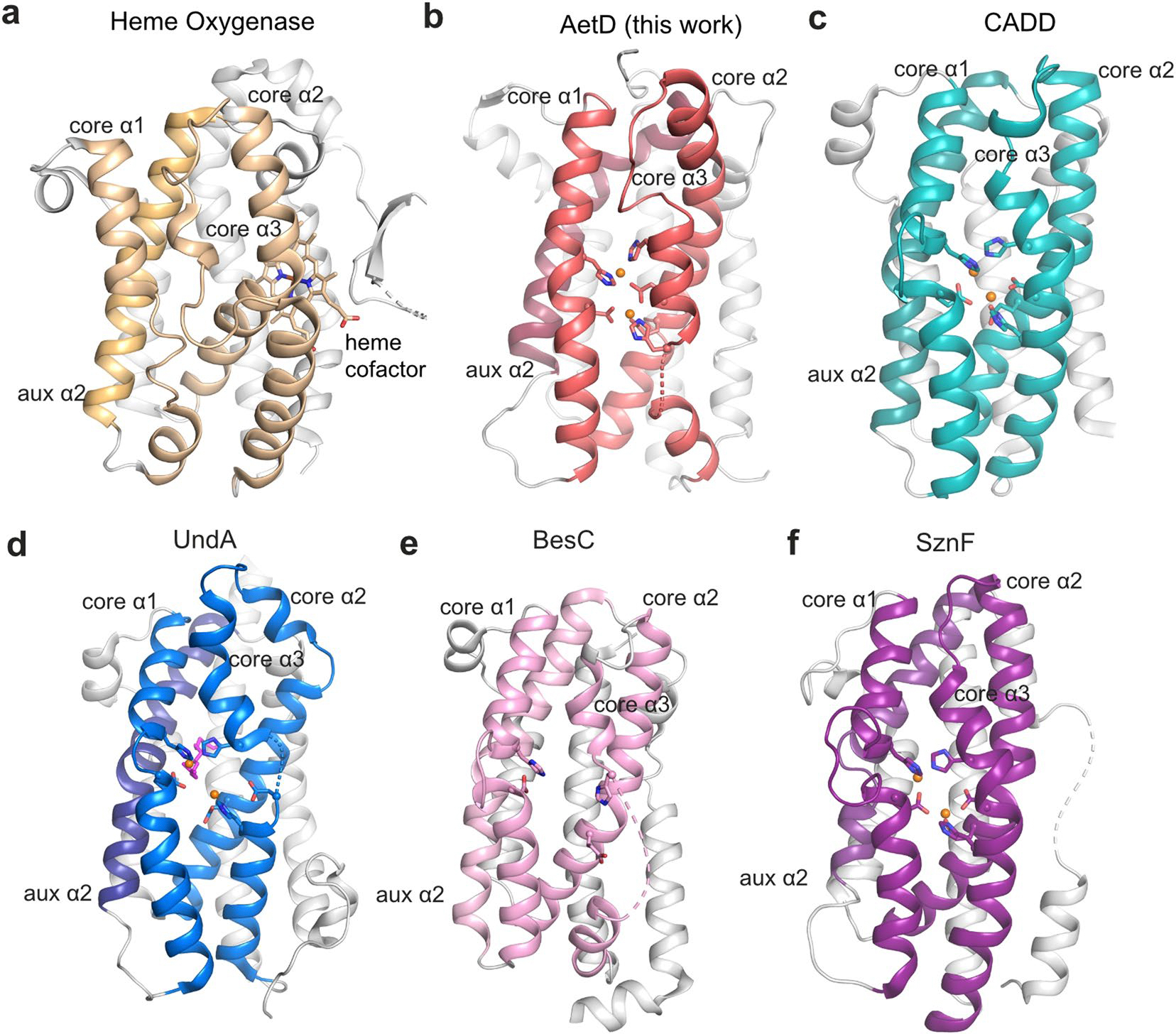
Structural comparison of AetD with haem oxygenase and other HDO enzymes. All HDO enzymes share the common architecture of three core helices containing metal-binding ligands, and four auxiliary helices. **a**, Haem oxygenase (PDB ID: 1WOW) **b**, AetD structure presented in this work. **c**, CADD (PDB ID: 1RCW). **d**, UndA (PDB ID: 6P5Q). **e**, BesC (PDB ID: 7TWA). **f**, SznF (PDB ID: 6VZY).

**Fig. 2 |:**
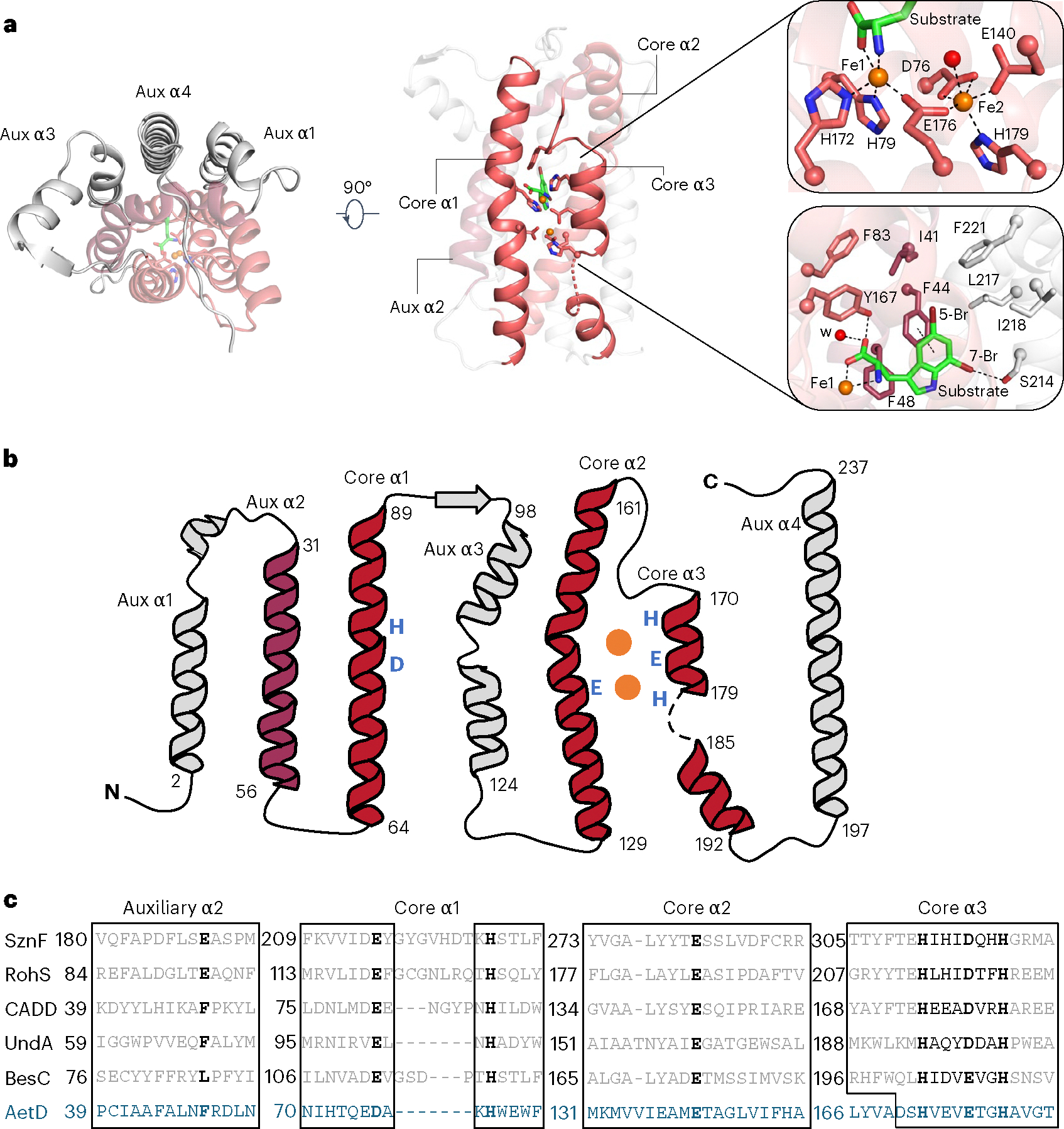
Structure and sequence alignments of substrate- and diiron-bound AetD with other HDO enzymes. **a**, Left: top-down view of holo-AetD. Middle: overall 7-helical bundle architecture of AetD. The three core α helices (cores α1, α2 and α3) that harbour metal-binding ligands (sticks) are shown as red ribbons. Auxiliary α2 (aux α2) is rendered in dark red. The rest of the auxiliary helices are coloured grey. Substrate 5,7-dibromo-l-tryptophan is represented by sticks (C, green; N blue; O, red; Br, brown). Upper right: diiron (orange spheres) cofactor site with interactions to Fe1 and Fe2 (occupancies 92% and 63%, respectively) indicated by dashed lines. Water molecules are represented by red spheres. The density for the diiron site is shown in Fig. 3d. Lower right: substrate binding site. Substrate is stabilized by hydrophobic interactions, coordination to Fe1, and hydrogen bonds, with dashed lines representing either Fe-ligand coordination (substrate to Fe1) or hydrogen bonds. Distances are given in Extended Data Fig. 3. **b**, Topology diagram showing the architecture of AetD. The six metal-binding residues are marked blue. The two orange circles represent the diiron cofactor. **c**, Sequence alignment of AetD with other enzymes in the HDO superfamily. The boxes show amino acid sequences that surround the conserved motifs within the α helices. The 3-His/3-carboxylate motifs (marked bold) are distributed into three core helices, a characteristic shared among HDO enzymes. One partially conserved residue in aux α2 is marked bold. This residue is a carboxylate ligand for non-substrate-triggered HDOs but a hydrophobic residue for substrate-triggered HDOs.

To investigate the structure of AetD and the mechanism of the AetD-catalysed reaction, we determined the crystal structure of substrate-bound AetD to 2.30 Å resolution via selenomethionine (Se-Met) single wavelength anomalous diffraction phasing (Supplementary Table 1). We then used that structure to determine the structure of substrate-bound AetD to the higher resolution of 2.08 Å, as well as two Fe(II)- and substrate-bound AetD structures to 2 Å and 2.30 Å, respectively (Supplementary Table 1). Despite low sequence homology according to BLAST and the enzyme function initiative-enzyme similarity tool EFI-EST, structural analysis demonstrates that AetD is a member of the HDO enzyme superfamily^29^. AetD seems to be dimeric (Extended Data Fig. 1); each AetD protomer has a 7-helical-bundle architecture, which comprises three core helices that house residues responsible for coordinating the diiron cofactor, and four auxiliary helices (Fig. 2). Three of the auxiliary helices (aux α1, α3 and α4) pack tightly against aux α2 and the core helices, forming a largely hydrophobic substrate binding pocket (Fig. 2a,b and Supplementary Fig. 1). This same 7-helical bundle fold was previously observed for the HDO enzymes CADD^30^, UndA^21^, BesC^28^ and SznF^25^ (Extended Data Fig. 2). Members of the HDO structural family differ from those of ferritin-like diiron oxidases/oxygenases (FDOs)^31^ in that HDO enzymes have very labile diiron cofactors, which has posed a challenge for attaining structures of these enzymes with both iron ions bound^17^.

**Extended Data Fig. 3 |:**
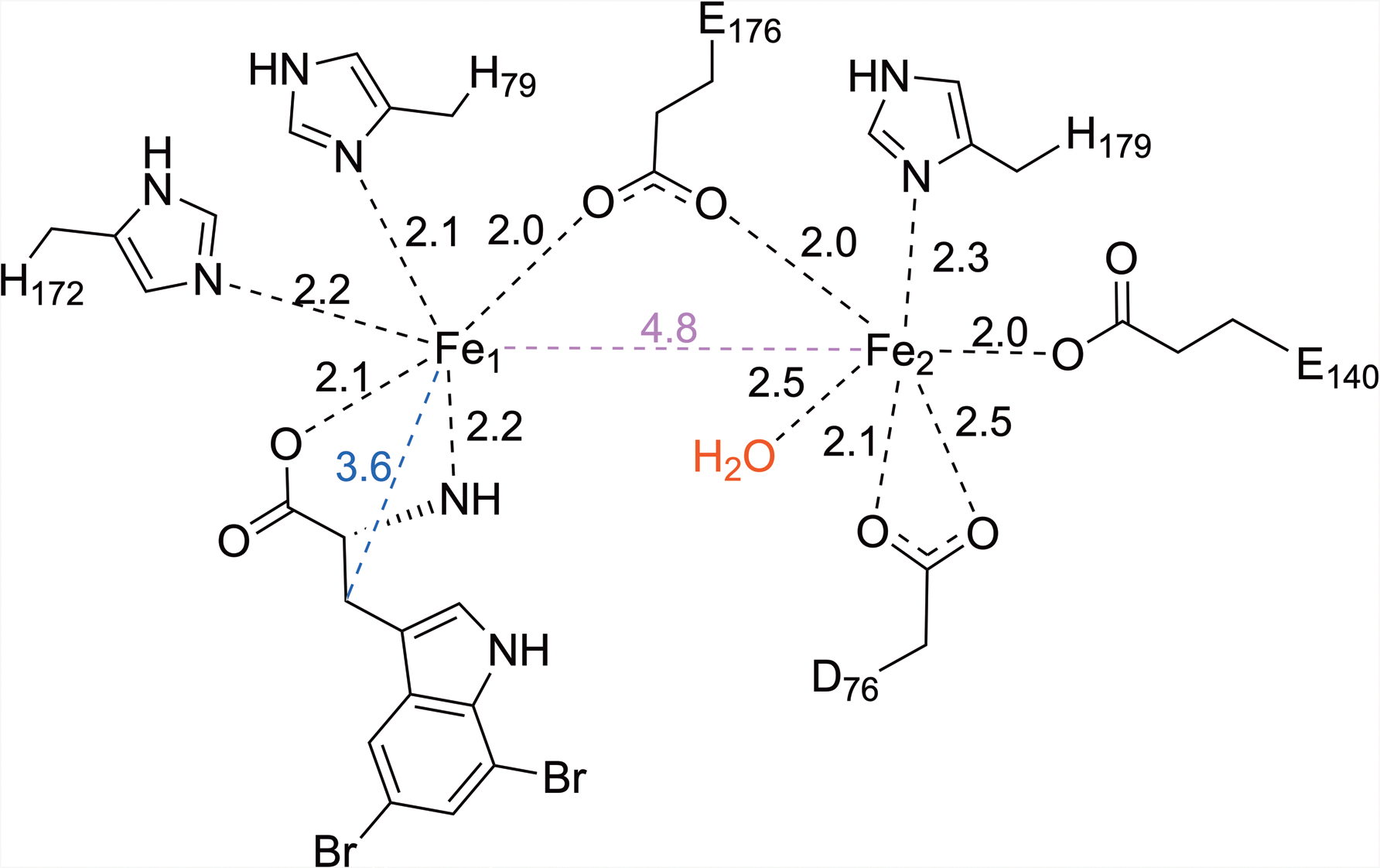
Schematic representation of the first coordination sphere of the diiron active site of Fe_2_(II/II)-AetD. Fe-ligand distances are in black dashes, Fe-Fe distance is in purple dash, and Fe-substrate (β-carbon) distance is shown in blue dash. All distances are in angstroms (Å).

**Extended Data Fig. 4 |:**
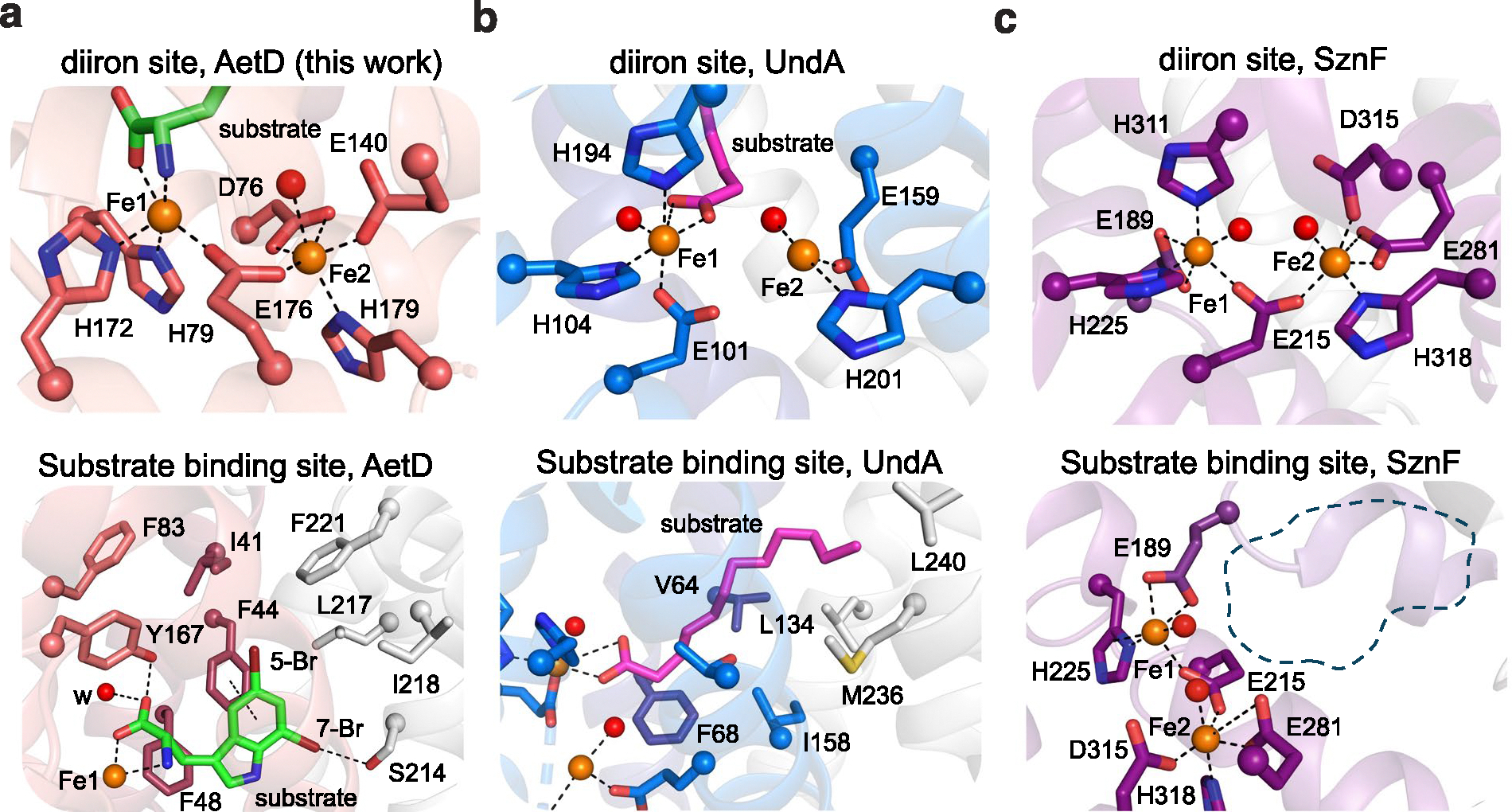
HDO structural comparison of substrate binding site and diiron cofactor sites. **a**, Diiron site and substrate binding sites of AetD reproduced here from Fig. 3A. Substrate shown with green carbons. **b**, Diiron site and substrate binding sites of UndA^25^ (PDB ID: 6P5Q) are similar to those of AetD. In both structures, the substrate is stabilized by hydrophobic interactions at enzyme core. AetD substrate binds Fe1 in a bidentate manner whereas UndA binds Fe1 only through one of the oxygen atoms in its carboxylate moiety. **c**, Diiron site and putative substrate binding site of HDO enzyme SznF^27^ (PDB ID: 6VZY). Note: no substrate-bound structure is available for SznF; the dashed line in the bottom panel indicates the expected substrate binding position.

An AetD structure reconstituted with iron shows two iron ions bound in a diiron cofactor, albeit with less than full occupancy (92% and 63% for Fe1 and Fe2, respectively); however, this structure does allow us to identify the residues involved in coordinating the diiron cofactor. In particular, the structure shows that that core helix α1 (nomenclature based on ref. 25) provides a monodentate ligand (H79) to Fe1 and a bidentate ligand (D76) to Fe2 (Fig. 2a, upper right, and Fig. 2b,c). Core helix α2 provides a single monodentate E140 ligand to Fe2, whereas core helix α3 presents a characteristic ^170^HX_3_EX_2_H^179^ motif that completes the coordination of the diiron cofactor (Fig. 2 and Extended Data Fig. 3). This metal binding site is similar to that of other HDO enzymes, such as CADD^23,30^, UndA^21,26,32^, BesC^22,27,28^ and SznF^18,24,25^ (Extended Data Figs. 2 and 4).

Interestingly, in these structures, the core helices often deviate from classical helical structures to appropriately position the metal-binding ligands. For example, the α1 helix in AetD, UndA^26^ and BesC^28^ has a kink at its halfway point, at which the helix contributes metal-binding ligands to the active site (Extended Data Fig. 2d,e). In CADD^30^ and SznF^25^, α1 is completely interrupted by a sizeable loop at this same location (Extended Data Fig. 2c,f). The core helix α3 is also unusual in that its C-terminus is highly flexible allowing the helix to unwind (Extended Data Figs. 1 and 2), presumably to provide access to substrates and/or cofactor binding sites^21,25,28^.

Structural comparisons suggest that there are differences across α1–3 and aux α2 that are probably important for substrate specificity. For example, core α3 starts later in the AetD amino acid sequence than in other HDO enzymes (Fig. 2c), which results in the α3 residue Y167 being directly placed into the active site, where it can form a hydrogen bond with the substrate carboxylate oxygen (Fig. 2a,c). Whereas the aux α2 helices in SznF and RohS contribute Fe1 ligands E189 and E93, respectively (Fig. 2c and Extended Data Fig. 4c), in AetD this residue is F48, and its side chain stacks against AetD’s substrate (Fig. 2a, lower right). The absence of a Glu ligand in AetD provides an open coordination site on Fe1 for ligation of the substrate carboxylate and amine nitrogen (Fig. 2a). This direct interaction between the polar end of the substrate and Fe1 has also been observed in the substrate-bound structure of the HDO enzyme UndA^26^ (Extended Data Fig. 4). In AetD, the substrate is also anchored into the active site by hydrophobic interactions—including a putative *π*–*π* interaction with F44 (centroid-to-centroid distance of 4 Å)—and a 3 Å O–H···Br hydrogen bond between S214 and the substrate’s 7-Br substituent (Fig. 2a, lower right, and Supplementary Fig. 1).

### Conformational changes in AetD accompany cofactor assembly

**Fig. 3 |:**
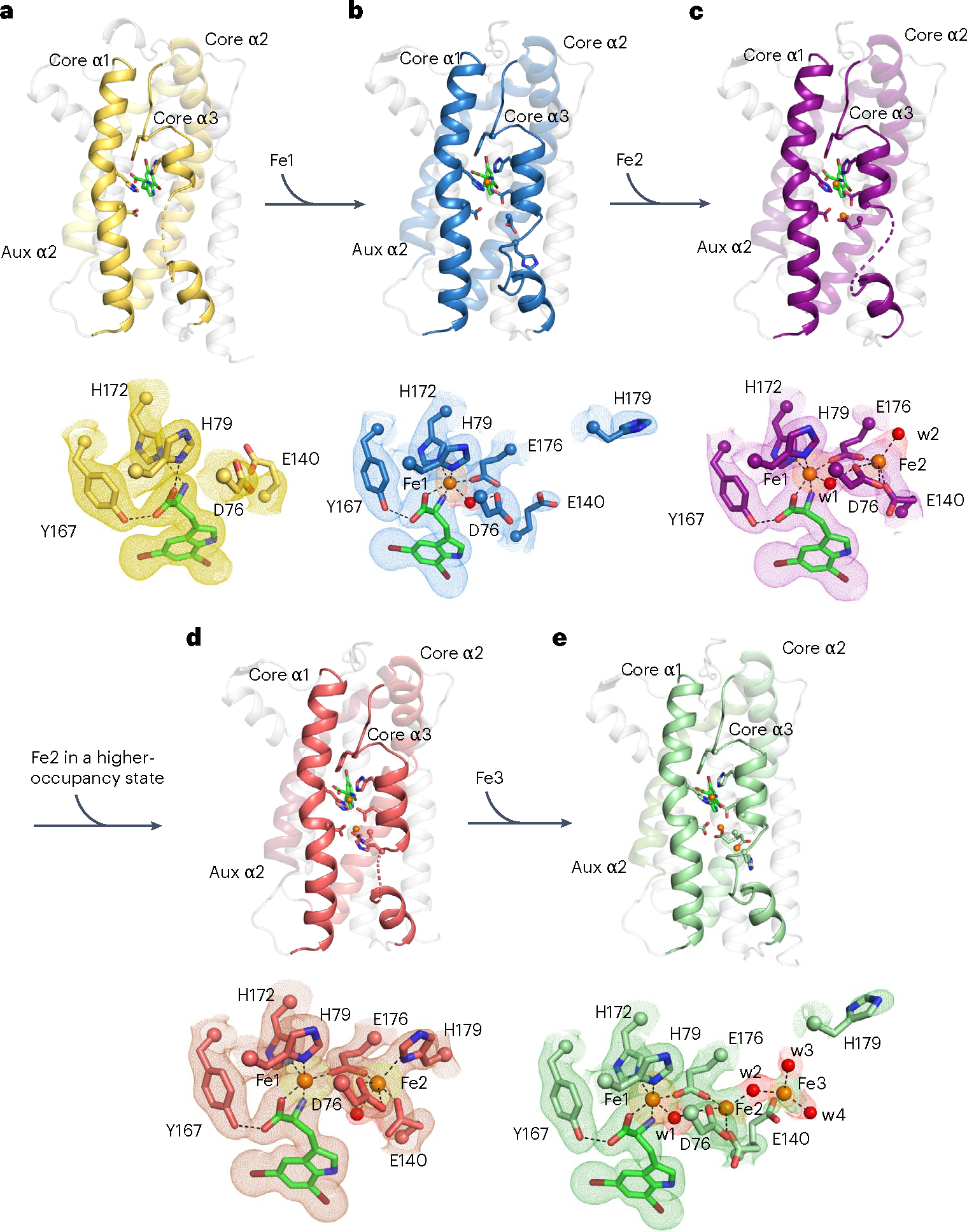
Crystallographic snapshots of substrate binding and diiron cofactor assembly. **a**, Only the substrate is bound; the cofactor is in a pre-assembly state. The amino acid moiety of the 5,7-dibromo-l-tryptophan substrate (green carbons) forms hydrogen bonds with Y167, and also with the Fe1 ligands H172 and H79. Core helix α3 only has one ordered helical turn. **b**, Substrate and Fe1 (orange sphere) are bound; the cofactor is in a partially assembled state. **c**, Substrate bound and cofactor assembled; Fe2 is in a low-occupancy state. **d**, Substrate and both irons are bound; the cofactor seems fully assembled, albeit with lower than full occupancies (92% and 63% for Fe1 and Fe2, respectively). **e**, Substrate bound and cofactor assembled, with a third iron bound at the cofactor. The substrates form similar interactions with the protein in all of the structures (omitted for simplicity). Selected amino acid side chains are represented by coloured sticks, with the α carbons of these residues represented by spheres. The water molecules are shown as red spheres. The 2*mF*_o_–*DF*_c_ composite omit maps contoured at 1*σ* are indicated by colored meshes in **a–e**. Iron anomalous difference maps are contoured at 3*σ* and are indicated by orange meshes in **a**–**c**,**e** and by a yellow mesh in **d**. The omit electron density of selected water molecules are indicated by red meshes.

The relationship between substrate binding and cofactor assembly is one of the more interesting features of HDO enzymes^24–28^. Diiron cofactors are typically stably bound by their enzymes and recycled in situ following each turnover event, but that does not seem to be the case for HDO enzymes, regardless of whether substrate binding triggers diiron cofactor assembly or not. Here, a series of crystallographic snapshots has allowed us to evaluate conformational rearrangements associated with diiron cofactor assembly in AetD. Yet, despite much effort, we were unable to obtain a structure of iron-bound AetD in the absence of substrate. However, we were able to obtain a substrate-bound co-crystal structure of AetD at 2.08 Å resolution (see Supplementary Table 1), which displays an excellent omit map density for 5,7-dibromo-l-tryptophan in the absence of iron (Fig. 3a and Supplementary Fig. 1). In this structure, the carboxylate and amino nitrogen of the substrate, which coordinate Fe1 when iron is present, now hydrogen bond to the Fe1 ligands H79 and H172, pre-organizing these residues for Fe1 coordination (Fig. 3a). Thus, the three ligands that exclusively coordinate Fe1 (the amino acid moiety of substrate, H79 and H172) are all positioned for Fe1 binding when the substrate is present. Diiron bridging residue E176, and Fe2 ligand H179, on the other hand, are part of a highly flexible region of core helix α3 (E176 to T183) and are disordered (Fig. 2 and Supplementary Fig. 2a). Of the residues that bind Fe2 (D76, E140, E176 and H179), only D76 of core helix α1 has good omit electron density (Fig. 3a). Substrate binding therefore seems to pre-organize AetD to bind Fe1 but not Fe2.

We obtained a snapshot of AetD with a single iron bound (Fe1) by expressing the protein in minimal media with Fe(II) supplementation, and then co-crystallizing the purified AetD with the substrate without any additional Fe(II). This substrate-bound structure with a partially reconstituted cofactor was determined to 2.3 Å resolution, with two protomers in the asymmetric unit (Supplementary Table 1). One protomer (chain A) has a single iron bound at high occupancy in the Fe1 site based on an iron anomalous difference map (Fig. 3b). The other protomer (chain B), described below, seems to have partial occupancy at both the Fe1 and Fe2 sites based on the iron anomalous difference map (Fig. 3c). First, for chain A, the single iron (Fe1) is coordinated by H79, H172, E176 and the substrate, as described above; D76 is positioned similarly to the substrate-only structure, but now makes a hydrogen bond with a water molecule that is also coordinated to Fe1, attaining an overall octahedral geometry. Core helix α3 is more ordered compared with the pre-cofactor assembly state (that is, substrate-bound-only state, Fig. 3a); there are two additional helical turns in the segment D170 to T177; T177 to I186 is now ordered as a non-helical loop (Supplementary Fig. 2a,b); and the Fe-bridging ligand, E176, and the Fe2 binding ligand, H179, are ordered adjacent to the Fe2 binding site (Fig. 3b). The density is improved for the Fe2 ligand E140, and, together with D76 and E176, these three residues form the Fe2 binding site (Fig. 3b).

Chain B of the partially reconstituted active site structure harbours two iron ions. When we set the B-factors of these iron ions in chain B to be the same as Fe1 in chain A—the iron ion with the lowest B-factor—and refine the occupancies, we find that Fe1 in chain B has a similarly high occupancy to Fe1 in chain A (~84%) with the same ligands and coordination geometry, and that Fe2 has approximately half of the occupancy of Fe1 (~43%). For this reason, we refer to this structure as partially reconstituted. Fe2 attains a square pyramidal geometry, coordinated by E176, D76, E140 and a water molecule (w2). The only missing ligand is H179, which is in the disordered segment (T177 to I186) of core α3 (Fig. 3c).

In an attempt to obtain a fully reconstituted diiron-bound AetD structure, we took crystals grown under the conditions used for partially reconstituted AetD and soaked them in a large excess (20 mM) of Fe(II). We obtained a 2 Å resolution structure, again with two protomers in the asymmetric unit (Supplementary Table 1). Based on iron anomalous difference maps, one protomer (chain B) has two sites occupied by Fe(II) (Fig. 3d), and the other protomer (chain A) has three sites occupied by Fe(II), as described below.

In chain B, the occupancy of Fe1 (92%) is still higher than for Fe2 (63%), but Fe2 now is now fully coordinated, revealing the coordination geometry of the diiron cofactor in AetD (Fig. 3d). H179 shows well-defined electron density and is clearly coordinated to Fe2 here. The rest of the coordination sphere for Fe2 is completed by the bridging carboxylate E176, the bidentate ligand D76, the monodentate ligand E140, and a water molecule *trans* to H179, resulting in an octahedral geometry for Fe2. Overall, the metal-binding region of core helix α3 (D170 to H179) adopts the most ordered backbone conformation (three helical turns) among all of our structures (Supplementary Fig. 2c). Beyond H179, however, the flexible region of core helix α3 is disordered. In chain A, the occupancy of Fe1 and coordination geometry are unchanged (Fig. 3e), but the occupancy of Fe2 is lower (~26%) and an alternative Fe(II) position (Fe3) is now visible at low occupancy (19%). Fe(II) ions are unlikely to be able to occupy both the Fe2 and Fe3 sites at the same time given that E140 can only coordinate one iron site at a time, and the presence of Fe(II) in the Fe3 site seems to flip H179 away so that it no longer ligates Fe2. Thus, the presence of an Fe(II) ion in the Fe3 site disrupts Fe(II) binding in the Fe2 site, and instead of representing a stable Fe(II) binding site, the Fe3 position may represent an entry position for iron ions to access the cofactor binding site from bulk solvent.

### Source of the carbon atom in the nitrile functional group

Our next step in delineating the AetD mechanism was to probe the individual fates of the 2-aminopropionate portion of the substrate 5,7-dibromo-l-tryptophan (**7**) to identify the carbon that is the origin of the nitrile group in **8**. Employing cell lysates of *Escherichia coli* (*E. coli*) expressing the *Salmonella enterica* tryptophan synthase (TrpS)^33^, we enzymatically synthesized three ^13^C-labelled isotopologues of tryptophan: [2-^13^C_1_]-l-tryptophan, [3-^13^C_1_]-l-tryptophan and [1,2,3-^13^C_3_]-l-try ptophan, using indole and labelled l-serine as the two building blocks. The single-component flavin-dependent halogenase AetF was then used to convert the ^13^C-labelled l-tryptophan moiety into the corresponding ^13^C-labelled substrate **7** for AetD^14^.

**Extended Data Fig. 5 |:**
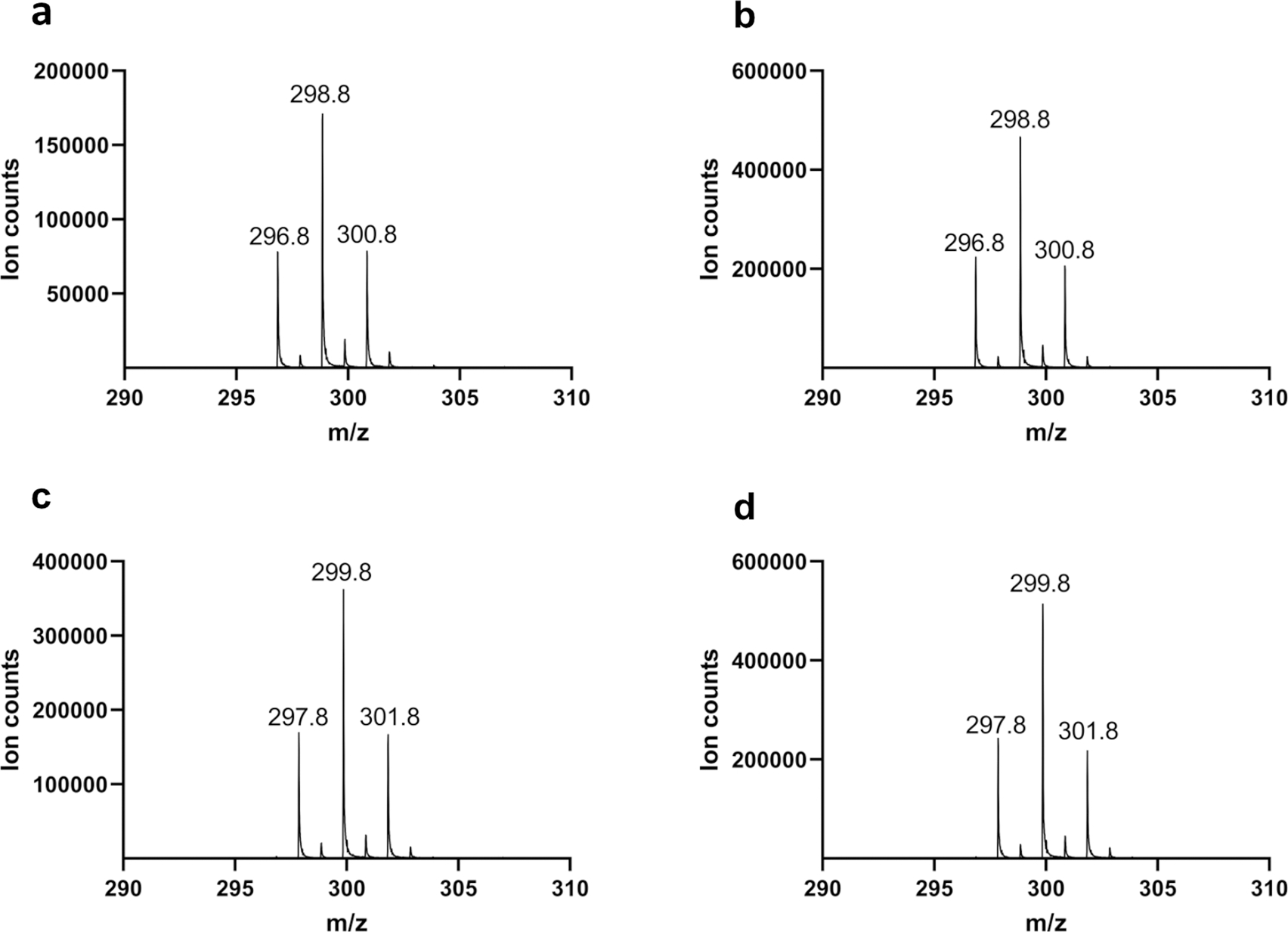
Mass spectra of the nitrile product when AetD reaction was performed with ^13^C-labelled isotopologues of 5,7-dibromo-L-tryptophan. **a**, AetD + 5,7-dibromo-L-tryptophan. **b**, AetD + [2-^13^C_1_]-5,7-dibromo-L-tryptophan. **c**, AetD + [3-^13^C_1_]-5,7-dibromo-L-tryptophan. **d**, AetD + [1,2,3-^13^C_3_]-5,7-dibromo-L-tryptophan. A 1 Da increase in the mass of the nitrile product was observed only when [3-^13^C_1_]-5,7-dibromo-L-tryptophan and [1,2,3-^13^C_3_]-5,7-dibromo-L-tryptophan were used as substrates, confirming the β-carbon C-3 is retained in the product.

**Fig. 4 |:**
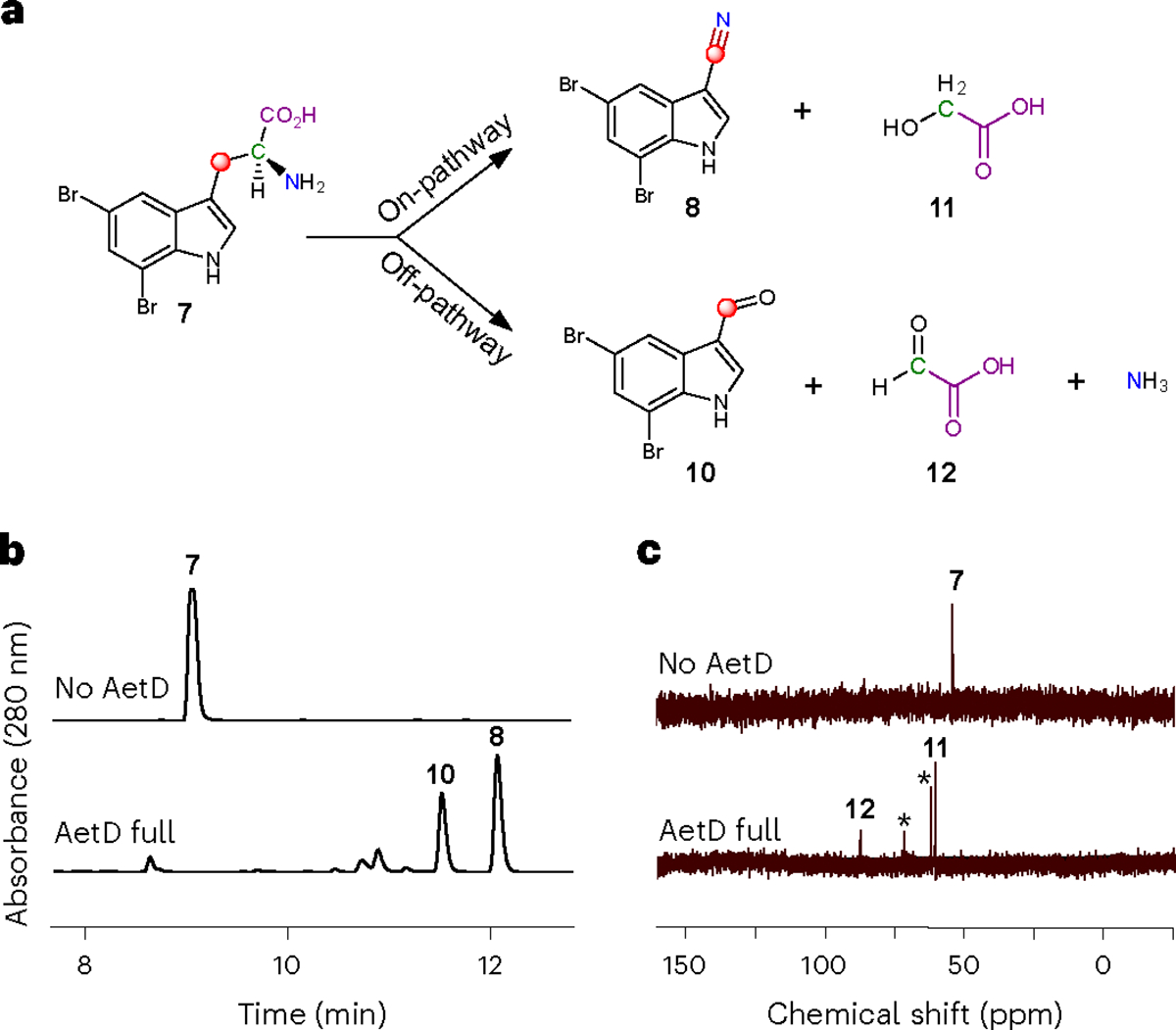
Characterization of all products in the AetD-catalysed reaction. **a**, Schematic showing the product distribution of the AetD-catalysed reaction in native and shunt pathways. The native pathway yields 5,7-dibromo-indole-3-carbonitrile (**8**) and glycolic acid (**11**) as products, whereas 5,7-dibromo-indole-3-carbaldehyde (**10**), glyoxylic acid (**12**) and ammonia are the products in the shunt pathway. Labelling experiments with ^13^C-labelled isotopologues of the substrate confirmed the fate of the carbon atoms of the 2-aminopropionyl side chain and are colour-coded accordingly. Refer to Extended Data Figs. 5, 7 and 8, and Supplementary Fig. 4 for full set of liquid chromatograms, and MS and NMR spectra. **b**, Liquid chromatogram showing the formation of shunt product **10** in addition to native product **8** in the AetD full reaction assay. **c**, ^13^C NMR spectrum of the AetD-catalysed reaction in the presence of [2-^13^C]-5,7-dibromo-l-tryptophan (**7**). In the full reaction assay, the C2 peak corresponding to substrate **7** disappeared, whereas two new ^13^C peaks appeared, which we characterized as the alcohol and aldehyde carbons of **11** and **12**, respectively. Asterisks represent glycerol peaks from the AetD protein stock.

Liquid chromatography–mass spectrometry (LC-MS) analysis of the reaction of AetD with the ^13^C-labelled 5,7-dibromo-l-tryptophans showed a 1 Da mass increase in the nitrile product only in the presence of [3-^13^C_1_]-5,7-dibromo-l-tryptophan and [1,2,3-^13^C_3_]-5,7-dibromo-l-tryptophan, establishing that C3 is retained in the nitrile product, whereas C1 and C2 are released (Fig. 4a and Extended Data Fig. 5). This result implies that the nitrogen atom attached to C2 must migrate to C3 to become the nitrile group, revealing an unusual rearrangement reaction.

### 5,7-Dibromo-indole-3-carbaldehyde is a shunt product

**Extended Data Fig. 6 |:**
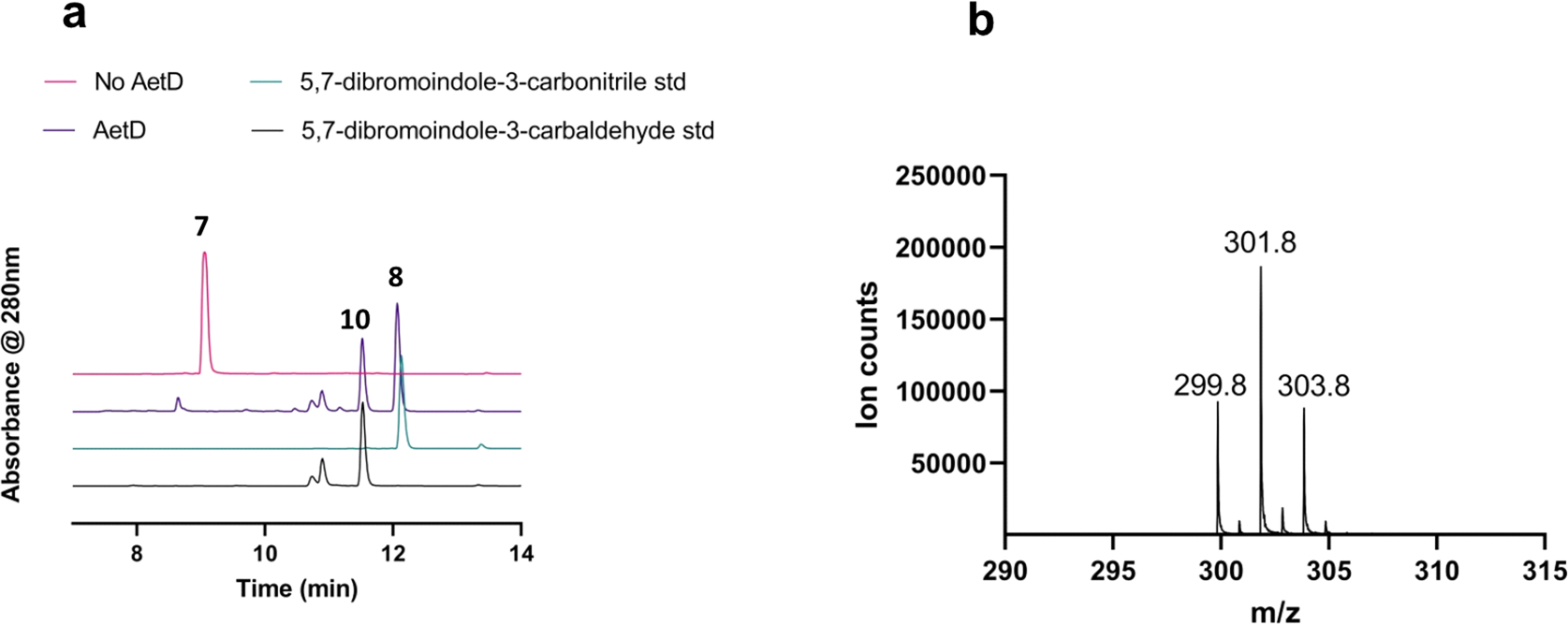
Characterization of the aldehyde shunt product formed in AetD reaction. **a**, HPLC chromatogram showing the formation of 5,7-dibromo-indole-3-carbaldehyde (**10**) product in addition to the native 5,7-dibromo indole-3-carbonitrile (**8**) product. The retention time of the aldehyde shunt product formed in the AetD-catalysed reaction matches with that of an authentic standard. **b**, The observed mass of 5,7-dibromo-indole-3-carbaldehyde product in negative ionization mode.

**Extended Data Fig. 7 |:**
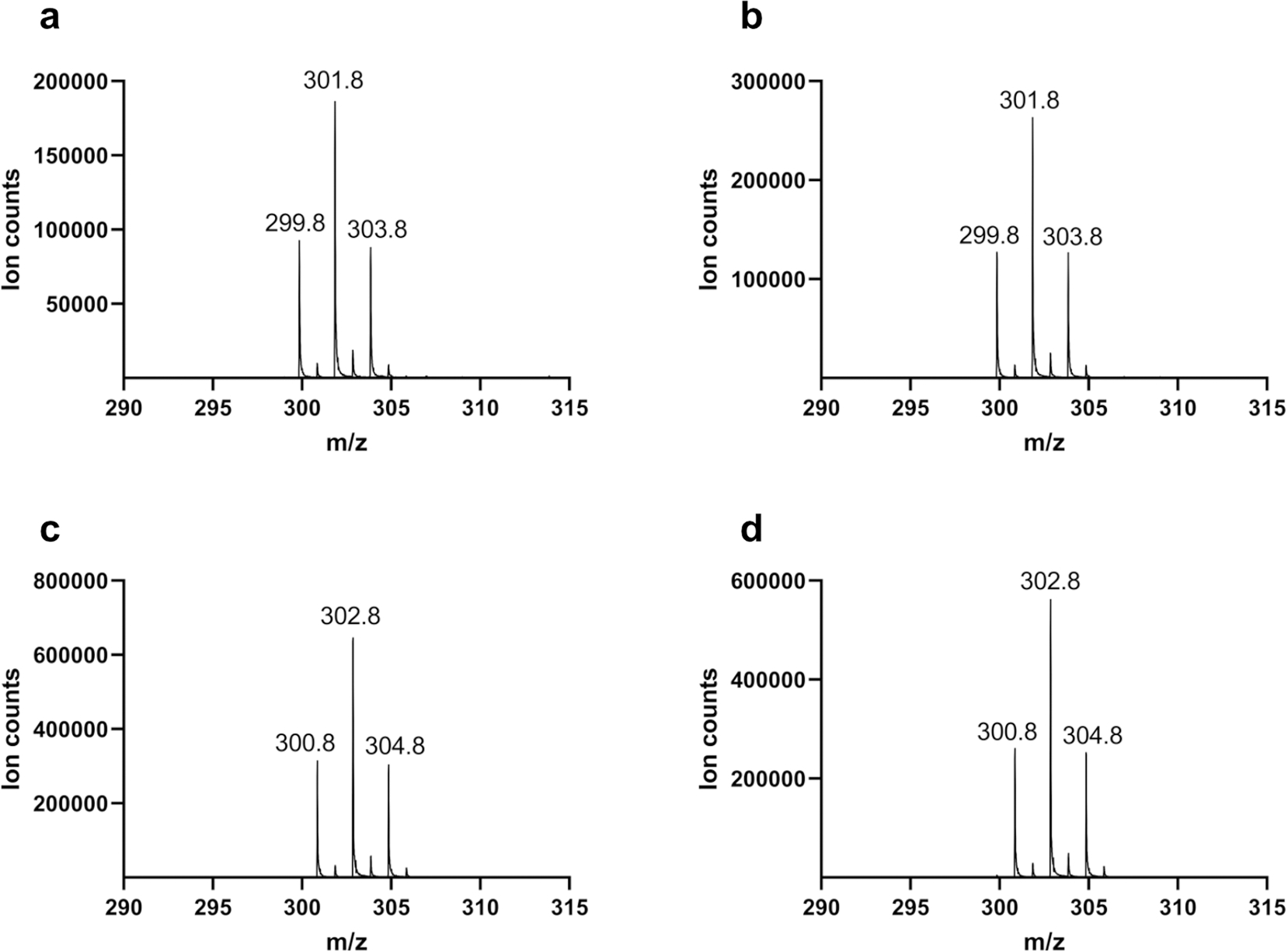
Mass spectra of the aldehyde shunt product when AetD reaction was performed with ^13^C-labelled isotopologues of 5,7-dibromo-L-tryptophan. **a**, AetD + 5,7-dibromo-L-tryptophan. **b**, AetD + [2-^13^C_1_]-5,7-dibromo-L-tryptophan. **c**, AetD + [3-^13^C_1_]-5,7-dibromo-L-tryptophan. **d**, AetD + [1,2,3-^13^C_3_]-5,7-dibromo-L-tryptophan. A 1 Da increase in the mass of the aldehyde shunt product was observed only when [3-^13^C_1_]-5,7-dibromo-L-tryptophan and [1,2,3-^13^C_3_]-5,7-dibromo-L-tryptophan were used as substrates, confirming the b-carbon C-3 is retained in the shunt product.

High-performance liquid chromatography analysis of the AetD reaction with 5,7-dibromo-l-tryptophan (**7**), however, revealed two product peaks (Fig. 4b). In addition to previously reported 5,7-dibromo-indole-3-c arbonitrile (**8**), we established 5,7-dibromo-indole-3-carbaldehyde (**10**) as a previously unreported second product. This assignment was confirmed via high-resolution mass data and comparisons with chemical standards (Extended Data Fig. 6). We incubated **10** in the presence of AetD along with ammonium salt or glycine to explore whether it could be further processed to the nitrile. Under these conditions, however, we did not observe any further transformation, suggesting that **10** is a shunt product and not an on-pathway intermediate. ^13^C-Labelling experiments revealed that C3 is retained in the aldehyde product like the native nitrile product (Extended Data Fig. 7). Although no previous biosynthetic routes exist for compound **10**, the des-bromo version of the compound has been previously reported^34–36^. To note, indole-3-carbaldehyde is produced by plants^34^ and human gastrointestinal microbiota^35,36^, and is proposed to be biosynthesized from tryptophan in a multi-enzyme-catalysed reaction. A more structurally similar compound, 6-bromo-indole-3-carbaldehyde, was reported from an *Acinetobacter* sp. bacterium associated with the ascidian *Stomozoa murrayi*^37^. However, the biosynthesis of this compound has not yet been reported.

### The fate of the released two carbon fragments

**Extended Data Fig. 8 |:**
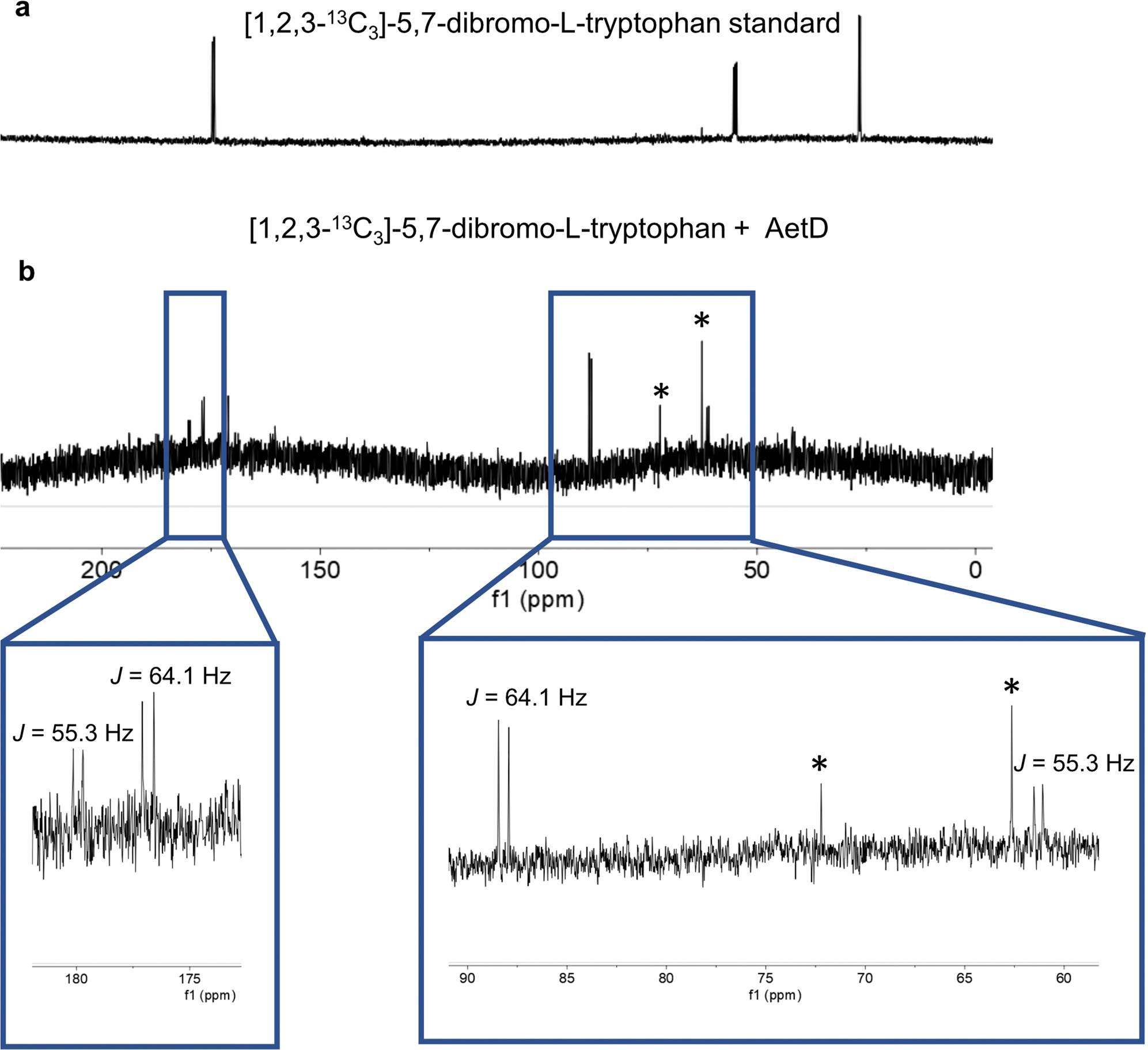
^13^C NMR experiment with [1,2,3-^13^C_3_]-5,7-dibromo-L-tryptophan. **a**, [1,2,3-^13^C_3_]-5,7-dibromo-L-tryptophan standard. **b**, [1,2,3-^13^C_3_]-5,7-dibromo-L-tryptophan + AetD enzyme assay. ^13^C-^13^C NMR J-couplings were observed for both glycolic acid (55.3 Hz) and glyoxylic acid (64.1 Hz) generated in the AetD-catalysed reaction. * Glycerol peaks from the AetD protein stock.

Having established the source of the carbon atom in both nitrile and aldehyde products **8** and **10**, we next explored the fate of the excised carbon atoms. Reaction of AetD with [2-^13^C_1_]-5,7-dibromo-l-tryptophan yielded two labelled product peaks in the ^13^C NMR spectrum: one at 61 ppm and the other at 88 ppm (Fig. 4c). These two peaks were assigned to glycolic acid (**11**) and glyoxylic acid (**12**), respectively, on the basis of their chemical shift values and comparison with standards (Supplementary Fig. 3). These assignments were corroborated by two further experiments. Reaction of AetD with [1,2,3-^13^C_3_]-5,7-dibromo-l-tryptophan gave rise to doubly labelled glycolic and glyoxylic acids, resulting in further splitting of the ^13^C NMR peaks due to ^13^C–^13^C *J*-couplings, thereby demonstrating that both carbon atoms originate from the same substrate molecule (Extended Data Fig. 8). The identity of the glyoxylic acid co-product was independently confirmed by *o*-phenylenediamine derivatization followed by mass spectrometry analysis (Supplementary Fig. 4). Comparison of the relative peak intensities in the ^13^C NMR and LC-MS spectra showed that **11** is the on-pathway side product, whereas **12** is a shunt pathway side product.

### μ-Peroxodiiron(III) accumulates in the AetD reaction

**Extended Data Fig. 9 |:**
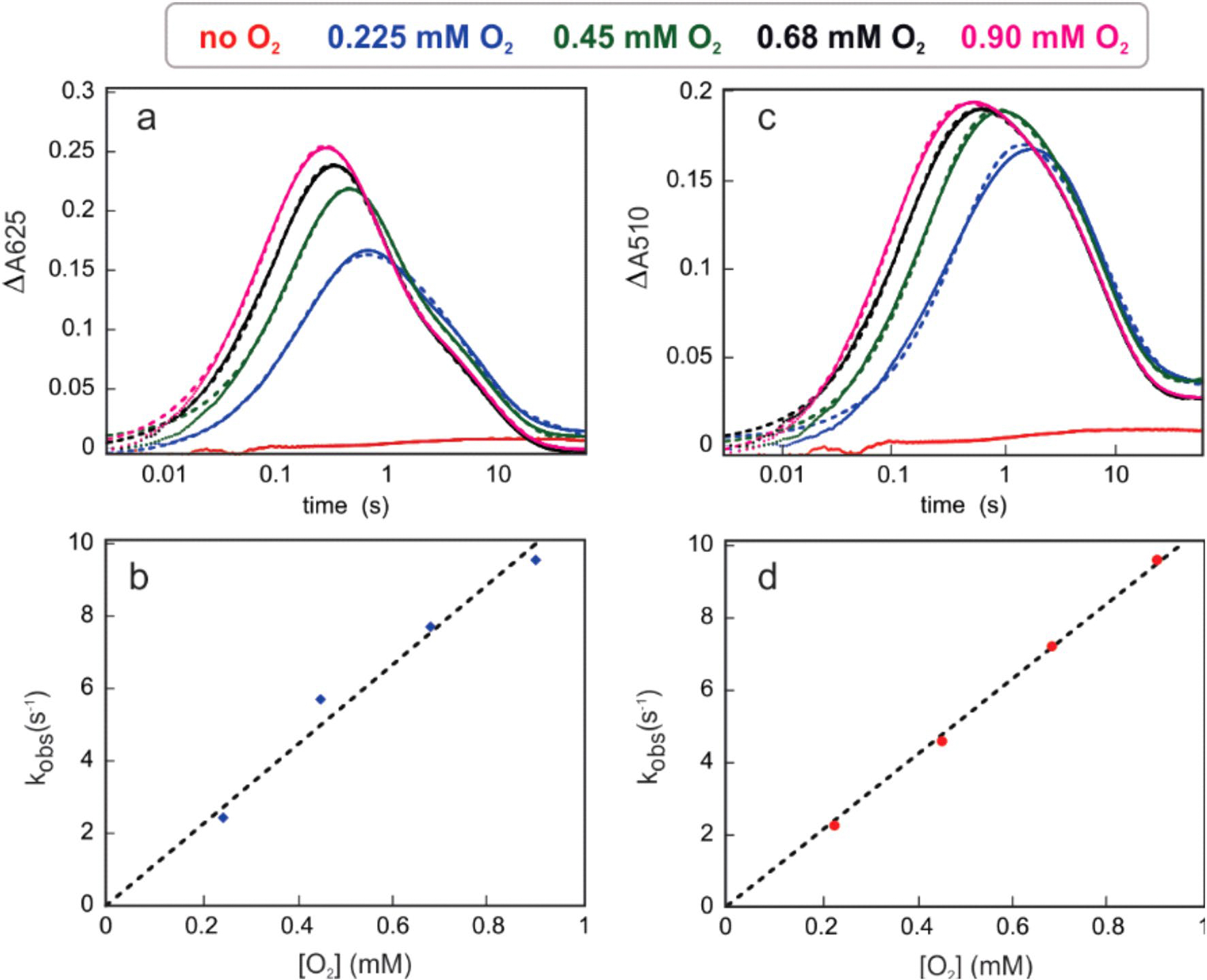
Kinetic traces showing the accumulation and decay of the absorbing intermediates. **a,b**, 625-nm absorbing intermediate as a function of time and O_2_ concentration **c,d**, 510-nm absorbing intermediate as a function of time and O_2_ concentration. Experimental conditions: O_2_-free solution AetD (0.30 mM), 5,7-dibromo-L-tryptophan (2 mM), Fe(II) (0.60 mM, 2 molar eq), temperature: 5 °C. Concentrations are reported prior to mixing (2-fold dilution).

**Fig. 5 |:**
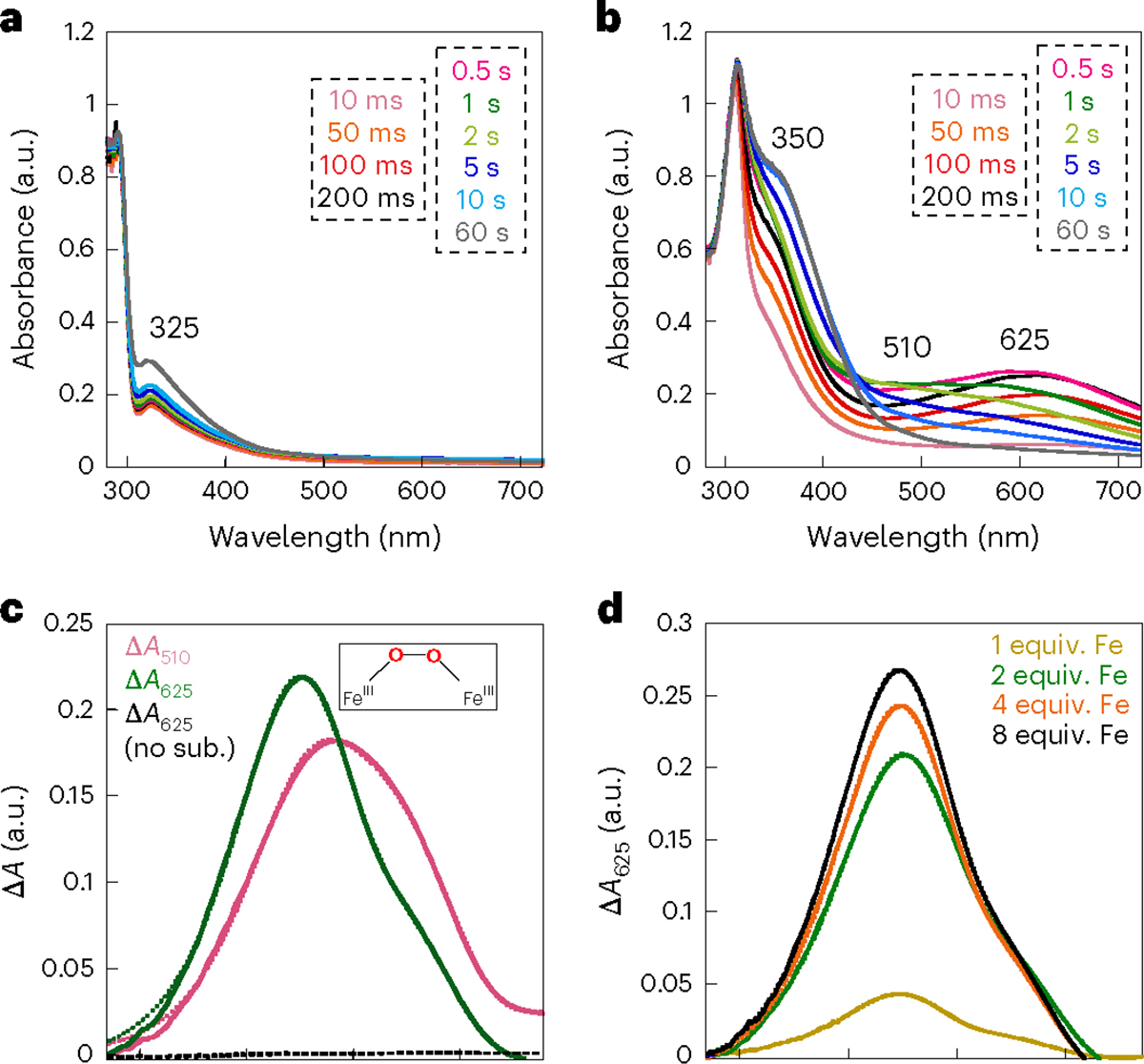
Stopped-flow-absorption spectra demonstrating the accumulation of intermediate(s) in the reaction of the Fe(II)·AetD complex with O_2_ in the presence of 5,7-dibromo-l-tryptophan at 5 °C. **a**,**b**, Absorption spectra acquired after rapid mixing an O_2_-free solution of AetD (0.30 mM) and Fe(II) (0.60 mM, 2 molar equiv.) in the absence (**a**) or presence (**b**) of 2 mM 5,7-dibromo-l-tryptophan, with an equal volume of O_2_-saturated buffer (1.8 mM). **c**, Kinetic traces showing the accumulation and decay of the absorbing intermediates as a function of time in the presence of 2 mM 5,7-dibromo-l-tryptophan (initial concentration). The control without the substrate is shown in black. **d**, Kinetic traces of the intermediate with absorption maxima *λ* = 625 nm as a function of various Fe(II) molar equivalents in the presence of 2 mM 5,7-dibromo-l-tryptophan (initial concentration).

The diiron HDOs characterized so far accumulate a transient peroxodiiron(III) species^24,26–28^ that has been shown to be an authentic intermediate in the case of BesC^28^ or SznF^24^. On this basis, we sought to observe any such intermediates in the AetD reaction. Reaction of an air-free solution of AetD and Fe(II) (2 molar equiv.) with O_2_-saturated buffer leads to the slow development (*k*_obs_ ≈ 0.06 s^−1^) of an absorbance feature with *λ*_max_ ≈ 325 nm, which signifies the oxidation of Fe(II) to Fe(III) (Fig. 5a). By contrast, when 5,7-dibromo-l-tryptophan is added to the ferrous solution of AetD and then reacted with O_2_, a new spectrum with absorption maxima at 350 nm and 625 nm rapidly develops (*k*_obs_ ≈ 8.2 s^−1^) (Fig. 5b), which exhibits a dependence on the concentration of O_2_ (*k*[O_2_] = 1.12 × 10^4^ M^−1^ s^−1^) (Extended Data Fig. 9). The absorption features of this species resemble those of μ-peroxodiiron(III) intermediates in non-haem diiron oxygenases and oxidases (that is, FDOs and HDOs), and are assigned to peroxo-to-Fe(III) charge transfer transitions^38^. The 625-nm-absorbing complex is transient and decays with an observed rate constant *k*_obs_ = 0.02 s^−1^ (Fig. 5c). The substrate-triggered accumulation of this species and its transient nature suggest that it may represent an intermediate in the reaction to yield the nitrile compound. Interestingly, we observed a second species (*λ*_max_ ≈ 510 nm) forming with a slower observed rate constant (*k*_obs_ = 7 s^−1^); the accumulation of which, however, is also dependent on the concentration of O_2_ (*k*[O_2_] = 1 × 10^4^ M^−1^ cm^−1^) (Extended Data Fig. 9). The absorption features of this second species are also consistent with a μ-peroxodiiron(III) species, for which the reported *λ*_max_ range is within 450–700 nm (ref. 38). Decay of the 625-nm-absorbing complex is not O_2_-dependent, unlike the formation of the 510-nm-absorbing species, suggesting that these two species represent separate events following the reaction of ferrous complexes with O_2_, which perhaps differ in the degree of substrate oxidation. The kinetics of the O_2_-dependence of the 625 nm species corroborates that no prior AetD-O_2_ adducts accumulate to a detectable level in the reaction. The 510 nm complex decays with accumulation of a ultraviolet–visible species at 350 nm that resembles that of μ-(hydr)oxodiiron(III) end reaction complexes^24,28^.

**Fig. 6 |:**
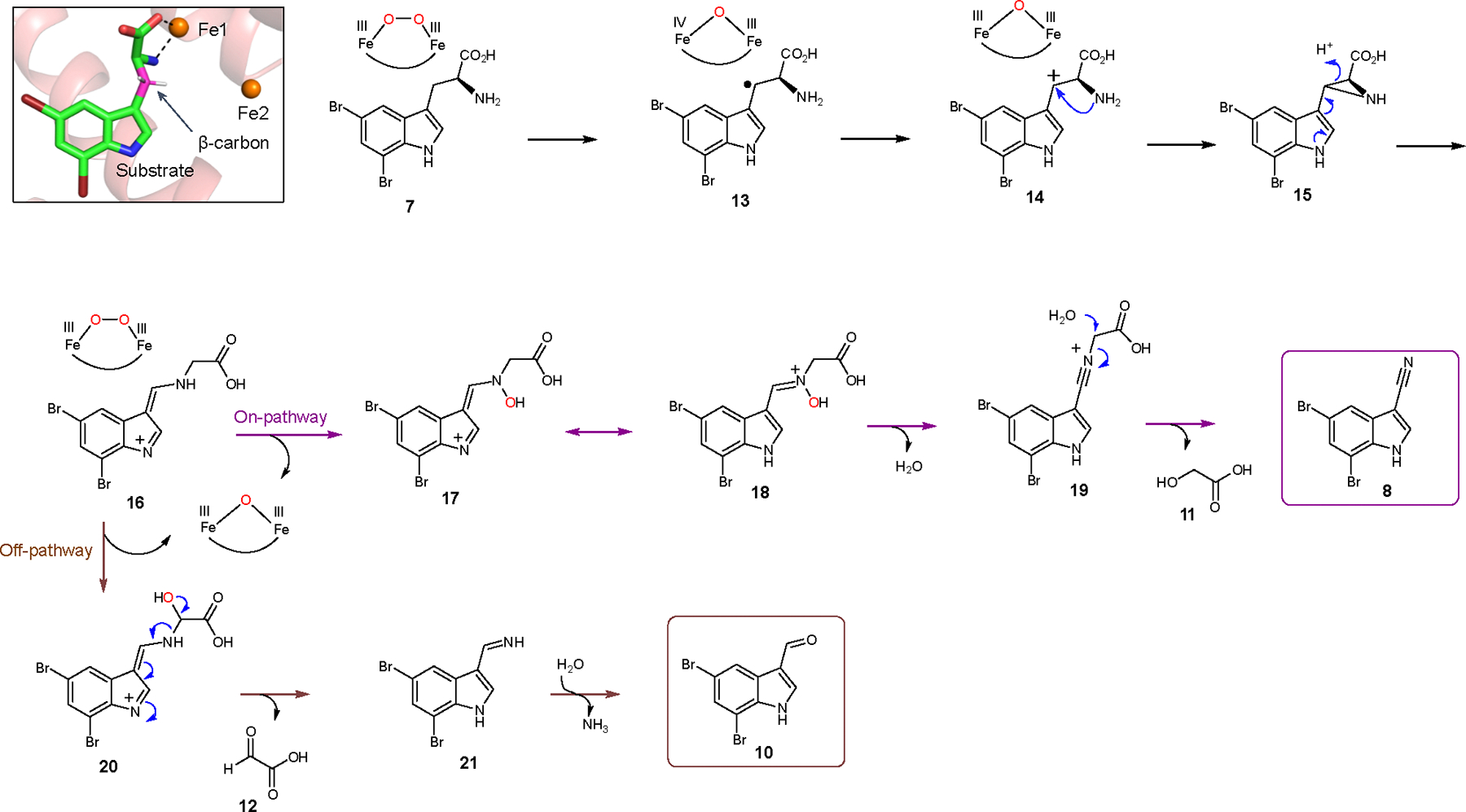
Proposed reaction mechanism of AetD-catalysed reaction. The top left panel is a magnified view of the active site of the substrate-bound Fe_2_(II/II)-AetD structure. The substrate β-carbon (coloured pink) is positioned for hydrogen abstraction by the peroxo-diiron intermediate. White sticks represent hydrogens on the β-carbon. Refer to Extended Data Fig. 10 for DFT calculations showing enthalpies and free energies associated with each individual step of a model pathway employing peracetic acid as an oxidant.

The kinetics of formation of the 625 nm and 510 nm transient species in AetD exhibits a similar dependence on Fe(II) concentration as previously observed for the μ-peroxodiiron(III) intermediate in BesC^27,28^. The addition of Fe(II) in excess stoichiometry leads to an increase in the apparent rate of formation of both species (Fig. 5d and Supplementary Fig. 5). This behaviour, which is suggestive of allosteric synergy between substrate and Fe(II) binding, is exhibited by both the 625 nm and 510 nm complexes in AetD. Although we have yet to obtain supporting kinetic and spectroscopic evidence, the 510 nm species may represent a second μ-peroxodiiron(III) intermediate proposed to mediate the later stage oxidation to yield the nitrile and carbaldehyde products (Fig. 6).

### Proposed reaction mechanism

Our structural and mechanistic studies allow us to propose that the AetD reaction proceeds via initial hydrogen atom abstraction at the β-carbon of 5,7-dibromo-l-tryptophan (Fig. 6). We find that direct coordination of the substrate to Fe1 positions the substrate's β-carbon within the diiron cofactor cavity, making the β-carbon a likely site of hydrogen atom abstraction (Fig. 6, inset). The transient absorption spectra of AetD are rich in features that are best explained by the formation of two peroxodiiron(III) species that are involved in the two oxidative events to yield the nitrile product. Assignment of these species is consistent with their optical features, kinetics, and O_2_- and Fe-dependence; however, their precise characterization, as well as their role in the oxidative steps, requires further studies via Mössbauer spectroscopy. Assuming that two peroxodiiron(III) species mediate conversion to the nitrile, we propose that the 625 nm species corresponds to the first μ-peroxodiiron(III) centre, and that itself or a higher-valent succeeding species^28^ abstracts the hydrogen atom from the β-carbon of the substrate to form compound **13**. In our scheme, we have included the peroxodiiron(III) intermediate as a potential species that performs the hydrogen atom abstraction, following that observed for BesC^28^. However, other mechanisms are plausible, including the possibility that a higher-valent species reversibly connected to the peroxodiiron(III) abstracts the substrate hydrogen atom. Loss of an electron gives the carbocation intermediate **14** that in turn is trapped by the amine group to form the aziridine intermediate **15**.

A similar aziridine formation mechanism has been reported in a Fe(II)/α-ketoglutarate-dependent oxygenase TqaL-catalysed reaction during the biosynthesis of 2-aminoisobutyrate in fungi^39^. Next, aziridine ring opening, in which the nitrogen atom is inserted between the α and β carbons, gives intermediate **16**. We believe that this intermediate is the branching point between on- and off-pathway reactions. The on-pathway reaction probably proceeds via a second μ-peroxodiiron(III)-mediated N-hydroxylation (510 nm species) to **17**, followed by tautomerization to yield the nitrone intermediate **18**. Formation of the second proposed μ-peroxodiiron(III) is probably afforded by either ascorbate or Fe(II), which allow for regeneration of the diferrous centre as well as the subsequent reaction with molecular oxygen (Supplementary Fig. 6). Formation of two peroxo(III/III) adducts is not entirely unprecedented and has been previously reported for the N-oxygenase SznF^24^, for which, however, these species are spectroscopically indistinguishable and not 'substrate’-triggered, in contrast to AetD. Loss of water to **19** allows for the re-addition of water at the α-carbon to release glycolic acid (**11**) and give the nitrile product **8**. In the shunt off-pathway, intermediate **16** rather undergoes C-hydroxylation to yield intermediate **20**. The C–N bond cleavage results in the formation of imine intermediate **21** and glyoxylic acid (**12**). Upon spontaneous hydrolysis, the imine gives 5,7-dibromo-indole-3-c arbaldehyde **10** as the shunt product.

**Extended Data Fig. 10 |:**
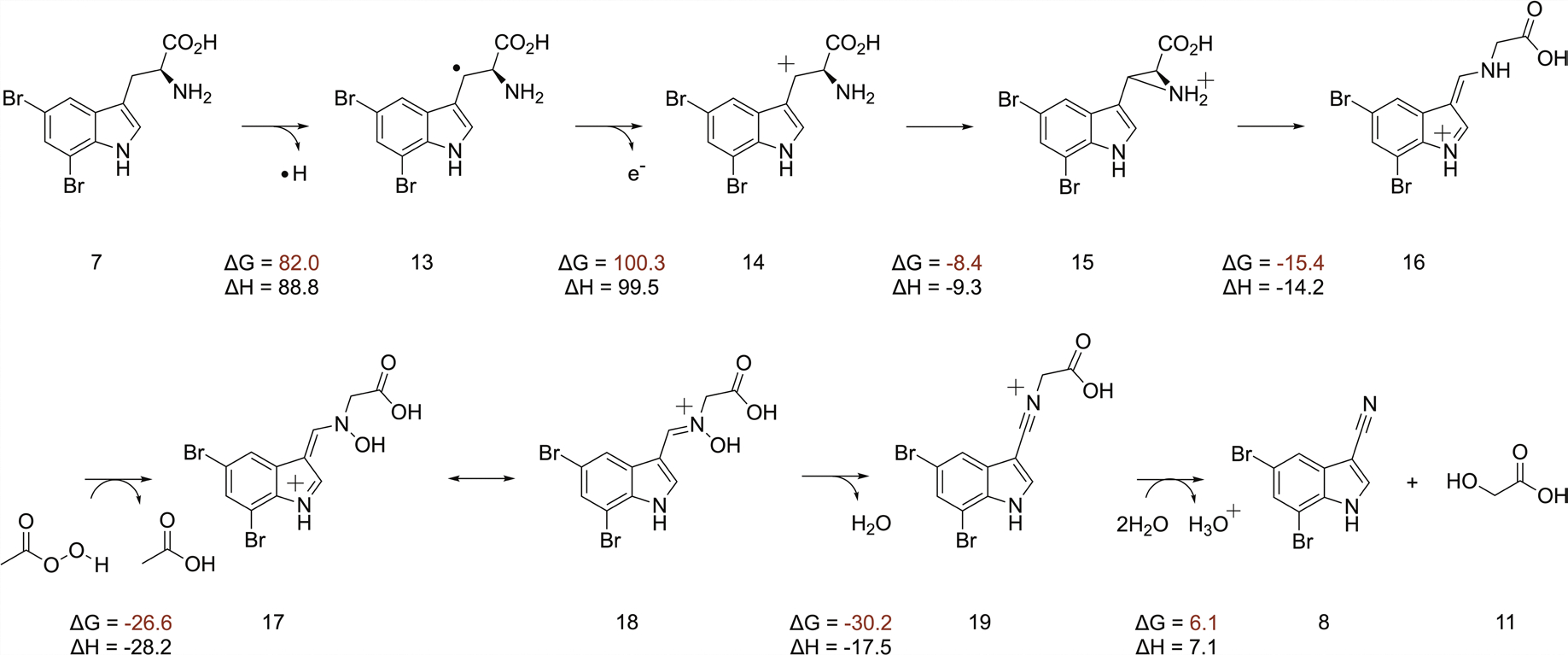
Density functional theory (DFT) calculations showing enthalpies and free energies associated with the individual steps in the proposed mechanism. The calculations are based on an approximation of the enzyme catalysed reaction as protein environment and diiron cofactor are not taken into account. Peracetic acid is employed as a substitute to simulate μ-peroxodiiron(III) mediated N-H oxidation.

To clarify the proposed mechanism, we further probed the initial hydrogen abstraction step. We synthesized the substrate with deuterium at the β carbon, [3-D_2_]-5,7-dibromo-l-tryptophan, by following our previously described chemoenzymatic method for the synthesis of ^13^C-labelled substrates. We then examined if the presence of deuterium at the β carbon of substrate led to a kinetic isotope effect (KIE) in the decay of the μ-peroxodiiron(III) species, as demonstrated in BesC^27,28^. We observed a KIE in the decay of the 625 nm species (Supplementary Fig. 7), but not in the 510 nm species. A KIE of 1.4 ∓ 0.3 for the 625 nm species is in the range of either a primary or secondary effect. The magnitude of the KIE, however, is lower than that previously reported for μ-peroxodiiron(III) intermediate decay in BesC, 2.1 ∓ 0.1, when the 4-position of substrate 4-Cl-lysine is deuterated^27,28^. The KIE value of 2.1 ∓ 0.1 for BesC was interpreted as an indication that the μ-peroxodiiron(III) intermediate was directly involved in hydrogen atom abstraction. Since it is hard to rationalize why a KIE would be observed if the hydrogen atom abstraction was performed by a subsequent intermediate to the μ-peroxodiiron(III) species, our proposed mechanism (Fig. 6) shows the reaction initially proceeding via the 625 nm μ-peroxodiiron(III) intermediate. More exhaustive experiments coupled to single-turnover product formation, similar to those done for BesC^28^ will further elucidate the role of the μ-peroxodiiron intermediates in AetD. We next performed density functional theory (DFT) calculations (Extended Data Fig. 10 and Supplementary Table 2). Although the formation of radical intermediate **13** and carbocation intermediate **14** are highly endergonic, there is a precedent of such chemistry in the BesC-catalysed reaction^27^. Subsequent steps involved in the formation of aziridine **15** and the rearranged intermediate **16** are exothermic. We employed peracetic acid as a substitute to simulate the μ-peroxodiiron(III)-mediated N–H oxidation, a step that is exergonic by −26.6 kcal mol^−1^ using this model oxidant. The final hydrolysis step is slightly endothermic with water as the nucleophile, but the enzyme active site probably increases the nucleophilicity of water and thus makes it more facile. Our proposed scheme best reflects our analytical, crystallographic and spectroscopic data, DFT calculations, and mechanistic precedents for oxidative chemistry by iron-containing enzymes. It should be emphasized that the mechanistic scheme we present in Fig. 6 is far from established, and that other avenues for nitrile formation are possible.

## Discussion and conclusion

AetD is a new addition to the HDO superfamily and performs a challenging rearrangement reaction yielding a nitrile product, providing an alternative route for nitrile biosynthesis. In terms of the mechanism of nitrile formation by AetD, our structural data suggest that substrate binds before or concomitant with diiron cofactor formation. In AetD, the substrate binding pocket is more accessible in the apo state when side chains are not positioned for metal binding and core α3 is unwound. Fe1 and Fe2 can access the cofactor binding sites through the unwound turn of core α3, which was also observed to be unwound before iron binding in the HDO enzyme SznF (Supplementary Fig. 8)^25^. It is only when core α3 is most ordered that the cofactor obtains its full complement of ligands with the highly flexible core α3 residue H179 providing the final ligation to complete cofactor assembly.

Our stopped-flow-absorption spectra support the prediction from the structure that the substrate binds before, or concomitantly with proper cofactor assembly, showing that the formation of the peroxodiiron(III) intermediate is substrate-triggered similar to BesC^28^ and UndA^26^. This observation is in contrast to SznF, for which spectroscopic data show that substrate binding is independent of dioxygen addition and peroxo-adduct formation^24^. The latter result for SznF suggests that proper diiron cofactor assembly can precede substrate binding, which could explain why a crystal structure of substrate-free diiron-SznF was attainable^25^ whereas a structure of substrate-free diiron-AetD was not. However, further studies are needed to fully understand the features that distinguish a substrate-triggered HDO from a substrate-independent HDO enzyme, and whether our AetD crystallographic data that show substrate binding occuring before iron binding are mechanistically sound, or represent a crystallographic artefact. While this manuscript was under revision, a paper reporting the crystal structure of AetD was published in which the authors similarly did not observe substrate-free diiron-AetD^40^.

Our overall mechanistic proposal for amine nitrogen migration to a β-carbon has literature precedents. Three different classes of aminomutases are known to perform such reactions^41^: adenosylcobalamin (B_12_)-dependent aminomutases (for example, lysine 5,6-aminomutase); *S*-adenosyl methionine (AdoMet)-dependent aminomutases (for example, lysine 2,3-aminomutase); and methylideneimidazole-5-one-dependent aminomutases (for example, phenylalanine aminomutase). In the case of the AetD-catalysed reaction, in addition to amine nitrogen migration to the β-carbon, there is also C_α_–C_β_ bond cleavage and further oxidation. A single-enzyme-catalysed C_α_–C_β_ bond cleavage reaction on tryptophan is rare, with the radical *S*-adenosyl methionine-dependent tryptophan lyase NosL being the lone example^42,43^. NosL catalyses a similar bond cleavage reaction during nosiheptide antibiotic biosynthesis while converting l-tryptophan to 3-methylindole-2-carboxylic acid. It is quite remarkable that AetD single-handedly performs both steps, which otherwise would have been a multi-enzyme-catalysed process.

In addition to their enzymatic capabilities, AetD and the other members of the HDO superfamily also exemplify the different ways in which nature tinkers with enzyme architectures (conformational gating) and substrate properties (metal-binding ability) to diversify and control enzymatic activities. Although we are at a nascent stage of discovering and exploring the wealth of the reactivities performed by HDOs, the results so far strongly argue that HDOs may be far more diverse with respect to structure and reactivity when compared with FDOs, which may be in part attributed to the apparent plasticity of their cofactors. We anticipate that this work will inspire future efforts of rational design and directed evolution on AetD and other enzymes in the HDO superfamily, by taking advantage of their cofactors and dynamic scaffolds to evolve unique chemical reactivities and/or expanded substrate scope. For example, engineering AetD to allow for the incorporation of nitrile functional groups into other amino acid substrates is an exciting direction towards expanding our inventory of nitrile-containing compounds.

### Online content

Any methods, additional references, Nature Portfolio reporting summaries, source data, extended data, supplementary information, acknowledgements, peer review information; details of author contributions and competing interests; and statements of data and code availability are available at https://doi.org/10.1038/s41557-024-01603-z.

## Methods

### Overexpression and purification of AetD

A pET-28a(+) plasmid containing the *E. coli* codon-optimized *aetD* gene between NdeI and XhoI restriction sites was synthesized and subcloned by Twist Bioscience. For the activity assay, AetD overexpression was performed in terrific broth media following the reported protocol^14^. For crystallographic and biophysical experiments, AetD overexpression was performed in M9 minimal media supplemented with 125 μM (final concentration) of ammonium iron(II) sulfate. In both cases, nickel affinity chromatography was used for the purification of the N-terminal His-tagged protein (sequence: MGSSHHHHHHSSGLVPRGSHM) following the reported protocol^14^. An additional round of purification was performed using size exclusion chromatography (Sephadex S200) on a fast protein liquid chromatography conducted on a Cytiva ÄKTA Pure 25 L1 system fitted with an F9-C fraction collector and S9 sample pump and controlled by Unicorn v.7 software. AetD was found to be dimer in solution based on the comparison of elution column volume with standards.

### Overexpression and purification of Se-Met AetD

A 125 ml sterilized flask with 50 ml sterile lysogeny broth was prepared. Kanamycin was added to the media with a final concentration of 40 μg ml^−1^. A single colony of *E. coli* BL21(DE3) harbouring the AetD-encoding gene in a pET-28a(+) vector was transferred to the media. The cell culture was grown at 37 °C with shaking at 220 r.p.m. for 16–20 h. The cell culture was pelleted by centrifugation at 10,000 g for 10 min at room temperature. The cell pellet was resuspended in 20 ml M9-kanamycin media. The cells were pelleted by spinning down. The above step was repeated to remove residual lysogeny broth. The pellet was resuspended in 1 l M9-kanamycin media. Cells were grown at 37 °C with shaking until OD_600_ ≈ 0.3. l-Lysine, l-phenylalanine and l-threonine were added to the culture with a final concentration of 100 mg l^−1^, whereas l-isoleucine, l-leucine, l-valine and l-selenomethionine were added to the culture with 50 mg l^−1^ final concentration. The culture was allowed to reach OD_600_ ≈ 0.6 when 0.5 mM IPTG was added. The induced culture was incubated at 15 °C (220 r.p.m.) for 20 h. Cells were harvested and protein was purified using nickel affinity chromatography following the reported protocol^14^. An additional round of purification was performed using size exclusion chromatography (Sephadex S200) on a fast protein liquid chromatography conducted on a Cytiva ÄKTA Pure 25 L1 system fitted with an F9-C fraction collector and S9 sample pump and controlled by Unicorn v.7 software.

### Overexpression of tryptophan synthase

A stock of *E. coli* BL21 (DE3) containing the overexpression plasmid of tryptophan synthase (pSTB7) was grown overnight in 10 ml lysogeny broth medium supplemented with 100 μg ml^−1^ of ampicillin; 10 ml of this overnight culture was used to inoculate 1 l of lysogeny broth medium containing 100 μg ml^−1^ of ampicillin. The cells were grown at 37 °C at 200 r.p.m. for 24 h. The cells were harvested by centrifuging at 10,000 g for 15 min. The cells were suspended in 35 ml of lysis buffer (100 mM KH_2_PO_4_, pH 7.5); 5 mg of pyridoxal 5’-phosphate was added, and four cycles of sonication were performed (each cycle is for 30 s with 1 s on/1 s off, 65% power) at 5 min intervals. The lysate was centrifuged at 30,000 g for 20 min at 4 °C and filtered through 0.2 μm filters to remove cell debris. The lysate was stored at 4 °C for up to one month and was used whenever required in this period.

### Stopped-flow protocol

Stopped-flow absorption measurements were performed in a SX20 stopped-flow spectrophotometer from Applied Photophysics, which is housed in an anoxic chamber (Coy Laboratories). All experiments were performed at 5 °C and were single-mix (two-syringe) experiments, in which the AetD reactant solution was twofold diluted. An O_2_-free solution of AetD (0.30 mM) that was reconstituted with two molar equivalents of Fe(II) (0.6 mM), with or without the dibromo-tryptophan substrate, was mixed with O_2_-saturated (on ice) 50 mM sodium HEPES buffer (pH 7.5) containing 10% glycerol, giving an estimated O_2_ concentration of 0.9 mM after mixing. All of the time-resolved absorption spectra were recorded using a photodiode array detector. In experiments in which we examined the dependence of the formation of the intermediate(s) as a function of iron equivalents, the O_2_-free AetD was mixed with different iron amounts before reaction with the O_2_-saturated buffer. The kinetics of formation and decay of the intermediates were fit by linear regression using:

$$\Delta A_{\text{int}\;}(t)=\frac{A\;k_{1}}{k_{2}\;-\;k_{1}}\left(e^{-k_{1}t}-e^{-k_{2}t}\right)$$

As the kinetic traces suggest that the intermediates exist in two forms, in most cases for fitting of the data we used the more expanded form to include an additional term accounting for the fact that the decay of the intermediates to the following state occurs with two different rate constants:

$$\Delta A_{\text{int}}(t)=\frac{A\;k_{1}}{k_{2}\;-\;k_{1}}\left(e^{-k_{1}t}-e^{-k_{2}t}\right)+\frac{A^{\prime }k_{1}}{k_{2}^{’}\;-\;k_{1}}(e^{-k_{1}t}-e^{-k_{2}^{\prime }t})$$

### Crystallization

All crystallization experiments were performed in an MBraun anaerobic chamber in an N_2_ environment. To prepare substrate-bound protein samples, 5 molar equiv. (1.75 mM) of enzymatically synthesized substrate 5,7-dibromo-l-tryptophan (see Supplementary Information ‘Methods’ for synthesis) dissolved in DMSO was added to 10.6 mg ml^−1^ (0.35 mM, measured by absorbance at 280 nm using an ultraviolet–visible spectrophotometer and an extinction coefficient of 35,410 M^−1^ cm^−1^, calculated using ProtParam^44^) AetD or 10 mg ml^−1^ (0.33 mM) Se-Met-labelled AetD in storage buffer (50 mM Tris-HCl, 100 mM NaCl, 10% v/v glycerol, pH 8.0), and then incubated overnight at 4 °C. The AetD protein had an uncleaved N-terminal purification tag with the sequence: MGSSHHHHHHSSGLVPRGSHM. The initial crystallization conditions for substrate-co-crystallized AetD were identified using crystallization screens dispensed by Mosquito liquid-handling robot (SPT Labtech) at room temperature. The identified conditions were then further optimized using the hanging-drop vapour diffusion method in the same anerobic, room-temperature settings. The substrate-bound structure was obtained from AetD expressed and purified from M9 minimal media without iron supplementation. The structure with a partially reconstituted metallocofactor was obtained from AetD expressed and purified from M9 minimal media supplemented with 125 μM (final concentration) ammonium iron(II) sulfate. The crystals that yielded these two structures grew under the following crystallization conditions: 100 mM 2-(*N*-Morpholino) ethanesulfonic acid monohydrate at pH 6, 20% w/v polyethylene glycol 4,000, and a salt mixture at pH 6 (110 mM malonic acid, 15 mM ammonium citrate tribasic, 7.2 mM succinic acid, 18 mM dl-malic acid, 24 mM sodium acetate trihydrate, 30 mM sodium formate and 9.6 mM ammonium tartrate dibasic). The structure with the fully reconstituted metallocofactor was obtained from the AetD expressed and purified from M9 minimal media without iron supplementation. The crystal grew under the following crystallization conditions: 250 mM ammonium sulfate and 20% w/v polyethylene glycol 3,350. These crystals were then soaked in 20 mM (final concentration) Fe(II) by adding to the drops an equal volume of well solution supplemented with 40 mM ammonium iron(II) sulfate and 10 mM substrate, which was then followed by overnight incubation with gentle mixing. All of the chemicals used for crystallization were purchased from Hampton Research. The hanging drops comprised 1 μl AetD or Se-Met-labelled AetD co-crystallized with substrate, and 2 μl of well solution from one of the two conditions above, in a sealed well over 500 μl of well solution. Transparent plate, rod or hexagonal-prism shaped crystals typically emerged overnight and grew to full size within one week. Crystals used for structure determinations were transferred to a Coy anaerobic chamber with an Ar/N_2_ gas mix environment for harvesting. The crystals were harvested, cryoprotected with either paraffin oil or the crystallization well solution supplemented with 20% v/v glycerol, and flash frozen in liquid nitrogen.

## Extended Data

## Supplementary Material

Supporting Information

Supplementary Data 1

## Figures and Tables

**Fig. 1 |:**
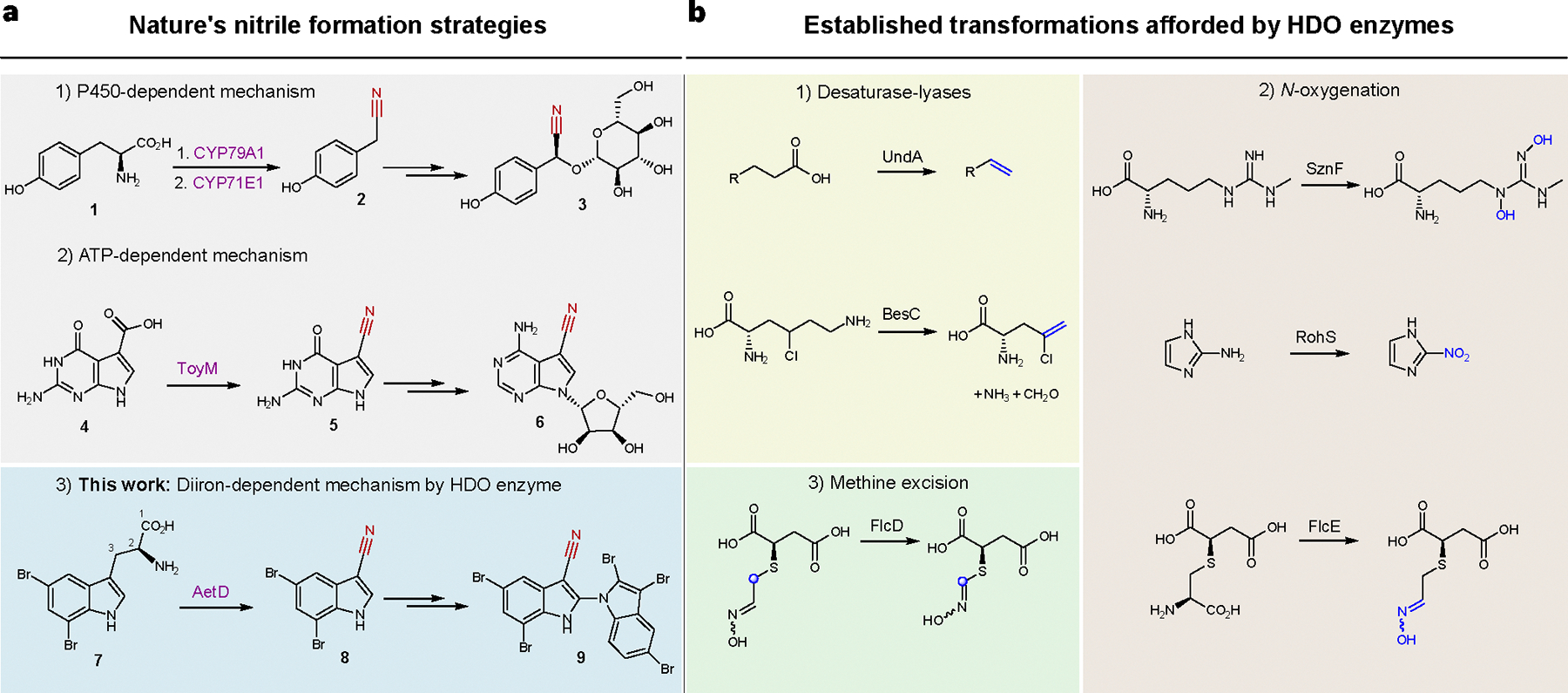
AetD is a member of the HDO enzyme superfamily that catalyses nitrile formation. **a**, Known nitrile biosynthetic enzymes in nature: (1) two cytochrome P450s are involved in converting tyrosine (**1**) via an oxime to nitrile **2** during the biosynthesis of the cyanogenic glucoside (*S*)-dhurrin (**3**); (2) ToyM catalyses the conversion of carboxylic acid **4** via an amide intermediate to nitrile **5** during toyocamycin (**6**) biosynthesis; (3) AetD converts the alanyl side chain of 5,7-dibromo-l-tryptophan (**7**) to nitrile **8** en route to the biosynthesis of cyanobacterial toxin aetokthonotoxin (**9**). **b**, Previously established reactions of HDO biochemistry. Nitrile formation expands the known reactivity of HDO enzymes, which includes (1) desaturase-lyases; (2) *N*-oxygenation; and (3) methine excision.

## Data Availability

The NCBI accession number of the AetD sequence used in this study is QNL15174. Atomic coordinates and structure factors for the crystal structures reported in this work have been deposited to the Protein Data Bank (PDB) under accession nos. 8TWN (substrate-bound AetD), 8TWT (substrate-bound AetD with diiron cofactor partially assembled) and 8TWW (substrate-bound AetD with diiron cofactor fully assembled). We also used the following PDB structures for AetD structural comparisons: 1WOW, 1RCW, 6P5Q, 7TWA, 6VZY, 6M9R and 6M9S. Other relevant data supporting the findings of this study are available in this published article or its Supplementary Information. Source Data are provided with this paper.
