# Supplementary material for: A single diiron enzyme catalyses the oxidative rearrangement of tryptophan to indole nitrile
Source: Nat Chem. 2024 Sep 16;16(12):1989–98. doi: 10.1038/s41557-024-01603-z (PMC11611611; doi:10.1038/s41557-024-01603-z)
Supplement: Supplementary file 1 — Experimental Protocol, Supplementary Figs. 1–9, and Tables 1 and 2. [file 41557_2024_1603_MOESM1_ESM.pdf]

nature chemistry

Supplementary information

https://doi.org/10.1038/s41557-024-01603-z

A single diiron enzyme catalyses the oxidative rearrangement of tryptophan to indole nitrile

In the format provided by the authors and unedited

Table of Contents

1. General Consideration 2
2. Preparatory HPLC parameters 2
3. LC-MS parameters 2
4. Enzymatic synthesis of 5,7-dibromo-L-tryptophan 3
5. Enzymatic synthesis of isotopologues of L-tryptophan 4
6. AetD reaction with ^{13}C labeled isotopologues of 5,7-dibromo-L-tryptophan 5
7. Derivatization of glyoxylic acid formed in AetD reaction using o-Phenylenediamine 5
8. X-ray data collection and processing 5
9. Structure determination and refinement 6
10. Computational methods 8
11. Supp. Fig. 1 Stereoview of substrate electron density 9
12. Supp. Fig. 2 Diiron cofactor assembly is conformationally gated by core helix α3 10
13. Supp. Fig. 3 ^{13}C-NMR experiments to identify the fate of the carbon fragments 11
14. Supp. Fig. 4 Detection of glyoxylate using o-phenylenediamine derivatization 12
15. Supp. Fig. 5 Kinetic traces of the intermediate with absorption maxima λ= 510 nm 13
16. Supp. Fig. 6 Dependence of multiple turnover activity on the presence of a reducing agent 14
17. Supp. Fig. 7 Kinetic traces of the intermediates in the presence of deuterated substrate 15
18. Supp. Fig. 8 Superimposition of AetD and SznF structures 16
19. Supp. Fig. 9 Characterization of [3-D_2]-5,7-dibromo-L-tryptophan 17
20. Supp. Table 1 AetD data collection and refinement statistics. 18
21. Supp. Table 2 Absolute calculation energies, enthalpies, and free energies 20
22. References 21

General Considerations

All chemicals were purchased from Sigma-Aldrich unless mentioned otherwise. LB broth (Lennox), TB broth, and kanamycin were purchased from Fisher Scientific. Ampicillin was from EMD Millipore. Potassium phosphate salt was purchased from Teknova. Amicon® Ultra-15 Centrifugal Filter Unit (10,000 MWCO) was obtained from Millipore. HisTrap column was obtained from GE Healthcare. Econo-Pack 10DG desalting columns were purchased from Bio-Rad. HPLC and LC-MS solvents were purchased from Fisher Chemical and were used without further purification. pET-28a(+) plasmid containing *E. coli* codon-optimized *aetD* gene between NdeI and XhoI restriction sites was synthesized and subcloned by Twist Bioscience. NMR spectroscopic data were obtained on a 500 MHz JEOL NMR spectrometer with a 3.0 mm probe. The values of the chemical shifts are described in ppm and coupling constants are reported in Hz. NMR data analysis was performed using MestreNova® 14.21-27684, 2021.

Preparatory HPLC parameters

Preparatory HPLC was performed with an Agilent Technologies system composed of a PrepStar pump, a ProStar 410 autosampler, a ProStar UV detector, and a fraction collector. A Phenomenex Luna C18 column (5µm, C18 100 Å, 100 x 21.2 mm) was used for separation. Data was collected and analyzed using Agilent OpenLAB CDS ChemStation software version C.01.05.

LC conditions:
A- water + 0.1% formic acid
B- acetonitrile + 0.1% formic acid

HPLC method: 10 mL/min flow rate; 0-5 min 5% B, 5-20 min 100% B, 20-25 min 100%B, 25-28 min 5% B, 28-30 min 5% B

LC-MS parameters

LC-MS analysis was performed with an Agilent Technologies 1260 Infinity series HPLC equipped with a degasser, binary pump, autosampler, and diode array detector coupled to an Agilent Technologies 6530 Accurate-Mass Q-TOF LC/MS. Separations were achieved using a

Kinetex 5 μm C18 100 Å, 150 x 4.6 mm column. Data was collected and analyzed using MassHunter Workstation Software version B.05.01.

LC conditions:
A- water + 0.1% formic acid
B- acetonitrile + 0.1% formic acid

LC method: 0.75 mL/min flow rate; 0-4 min 10% B, 4-11 min 10-95% B, 11-21 min 95% B, 21-23 min 95%-10% B, 23-27 min 10% B. Dual ESI ion source, negative ionization mode.

Enzymatic synthesis of 5,7-dibromo-L-tryptophan

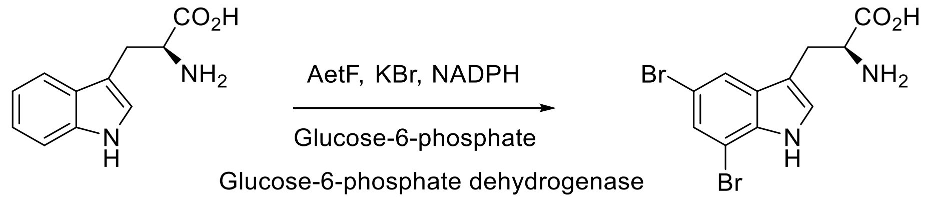

We closely followed our previous biocatalytic synthesis of 5,7-dibromo-L-tryptophan[1] with some minor adjustments. A 10 mL AetF reaction was prepared with 10 μM AetF, 1 U/mL glucose-6-phosphate dehydrogenase, 10 mM glucose-6-phosphate, 1 mM NADPH, 5 mM L-tryptophan (10 mg total), 50 mM KBr, in 100 mM KP_i pH 7.5 buffer. The reaction was incubated at 30 °C, 200 rpm overnight. The reaction mixture was lyophilized overnight and extracted 3 times with 1 mL methanol. The extraction was filtered through an 0.2 μm syringe filter and concentrated to ~500 μL by rotary evaporation. The 5,7-dibromo-L-tryptophan product was purified by preparative HPLC following the protocol described above. The typical yield was 7-8 mg. [3-D_2]-5,7-dibromo-L-tryptophan was synthesized following a similar protocol starting with [3-D_2]-L-tryptophan and the structure was validated based on HRMS and ^{1}H-NMR (See Supplementary Figure 19).

Enzymatic synthesis of isotopologues of L-tryptophan

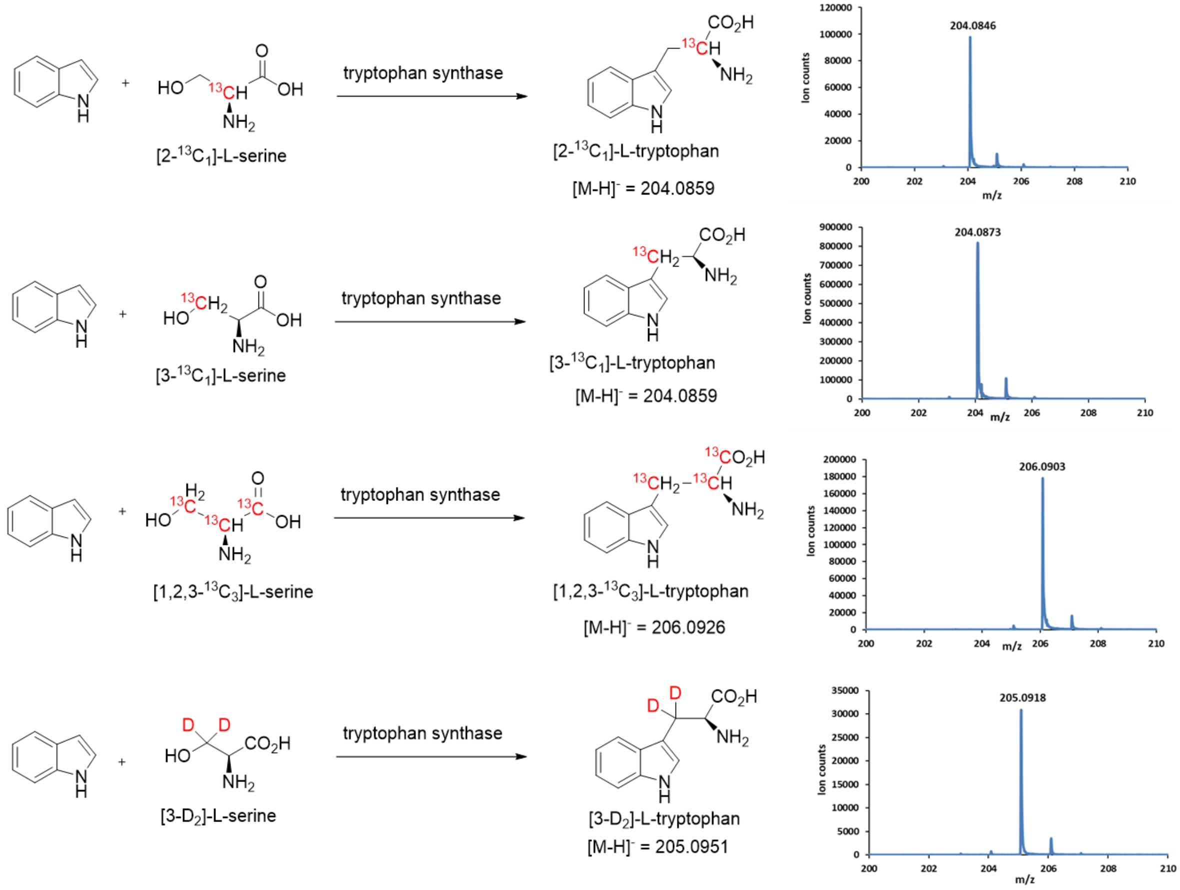

Isotopologues of L-tryptophan were produced following the reported protocol[2] with slight modification. 10 mg of indole and 12 mg of the respective labeled isotopologues of L-serine were dissolved in 10 ml of 100 mM potassium phosphate buffer (pH 7.5). 1 mL of cell lysate was added to initiate the enzymatic reaction. The reaction mixture was incubated at 37 °C at 200 rpm for 3 h. After the completion of the reaction, the reaction mixture was lyophilized, and the product was redissolved in methanol. Proteins were removed by ultrafiltration using 10 kDa cut-off filters (American Chromatography Supplies). Enzymatic products were purified on preparative HPLC using the protocol described above. The typical yield was 5-7 mg. The identity of the isotope-labeled tryptophans was confirmed based on LC retention time (compared with standard tryptophan) and HRMS.

AetD reaction with ^{13}C labeled isotopologues of 5,7-dibromo-L-tryptophan

100 µL reaction consisting of 20 µM AetF, 2 mM ^{13}C-labeled L-tryptophan, 5 mM NADPH, 20 mM KBr, 1 U/mL glucose-6-phosphate dehydrogenase, 5 mM glucose-6-phosphate in 100 mM KP_i pH 7.5 buffer was prepared in 1.5 mL centrifuge tubes and incubated at RT for 6 h. Then AetD, Fe (II), and ascorbate were added to the reaction mixture with final concentrations of 250 µM, 5mM, and 5mM respectively and the final volume of the reaction mixture was 200 µL. The resulting reaction mixture was incubated at RT overnight. The samples were analyzed using LC-MS and ^{13}C-NMR spectroscopy. For ^{13}C-NMR experiments, the samples were transferred to a 3mm NMR tube after adding 10% D_2O.

Derivatization of glyoxylic acid formed in AetD reaction using o-phenylenediamine

A 100 µL reaction mixture containing 500 µM 5,7-dibromo-L-tryptophan, 40 µM AetD, and 5mM Fe (II) was incubated at RT overnight. Next, the assay solution was made acidic (pH=1) by adding HCl. Then, o-phenylenediamine was added to a final concentration of 1 mM. The assay mixture was kept at room temperature for 2h. The reaction was quenched by adding 1:1 methanol, and the resulting solution was filtered using 0.2 µM centrifugal filters. The samples were analyzed by LC-MS.

X-ray data collection and processing

X-ray diffraction data were collected at cryogenic temperature (100K) at the Advanced Photon Source (Argonne, IL) beamline 24-ID-C of the Northeastern Collaborative Access Team (NE-CAT) using an Dectris Eiger-2 X 16 M detector, and at the Stanford Synchrotron Radiation Lightsource (Menlo Park, CA) beamline 9–2 using a Dectris Pilatus 6 M detector. The inverse-beam method was used to collect Fe and Se peak data in six 60° wedges to capture anomalous signals. All data were indexed, integrated, and scaled in XDS,[3] with data collected at the Fe and Se peak wavelengths processed anomalously with Friedel pairs kept separate. Resolution cutoffs were determined based on considerations of R_{sym}, $CC_{1/2}$, I/σ and data completeness in the highest resolution bin. Data collection statistics are summarized in Table S1.

Structure determination and refinement

The initial phases were obtained experimentally from a Se-Met AetD crystal (grown in the first crystallization condition) via single wavelength anomalous dispersion (SAD) method using data collected at Se peak (0.9791 Å). The Se anomalous signal extends to 2.6 Å resolution, where it has a $CC_{1/2\text{-anom}}$ of 0.5 as calculated by Phenix Xtriage.[4] Phenix Hybrid Substructure Search (HySS)[5,6] was used to find the positions of 10 Se sites out of 10 predicted sites for the two molecules in the asymmetric unit (ASU) (excluding the two Met residues that are part of the N-terminus purification tag, see sequence above). Refinement of heavy-atom sites and SAD phasing were performed by Phenix Experimental Phasing (Phaser-EP).[7] An initial experimental map was obtained to 2.8 Å resolution by Phaser-EP with a figure of merit (FOM) of 0.467 before any density modification. This initial map was then subjected to density modifications by Phenix RESOLVE[8] for solvent flattening and two-fold non-crystallographic symmetry (NCS) averaging, with phases extended to 2.3 Å resolution. An AlphaFold2[9]-generated AetD model was used to generate the mask for NCS averaging. The density-modified experimental map was easily interpretable, providing clear and contiguous electron density for manual model building in Coot.[10] This initial unrefined model in space group $P2_12_12_1$ was used to solve the two other structures of AetD in the same $P2_12_12_1$ space group, i.e. the structures of AetD with a fully or partially reconstituted cofactor, starting with rigid-body refinements in phenix.refine. 5% of total reflections from the AetD cofactor partially reconstituted dataset were randomly selected and set aside for the test set (R_{free}). This test set was carried over for the refinement of the Fe(II) fully-reconstituted structure. The substrate-bound-only structure was solved by molecular replacement (MR) in phenix.phaser using one chain of the initial unrefined model of the Se-Met dataset as the search model without further modifications. A new set of R_{free} was generated for this dataset using 5% randomly selected reflections. Iterative rounds of positional and individual B-factor refinements were performed in phenix.refine, with manual adjustments in Coot against $2|F_o|–|F_c|$ maps contoured to 1.0σ For the structures solved in space group $P2_12_12_1$ with two molecules in the ASU (see Table S1), two-fold NCS restraints were used throughout the refinements. Structure and geometric restraints for the substrate molecule were generated using eLBOW.[11] $2mF_o$-DF_c composite omit maps were generated, with 5% of the model omitted for individual omit maps, using Phenix Composite_omit_map[12] after each round of refinement to verify the refined structure. Water and other solvent molecules from crystallization conditions were added manually throughout model building using $|F_o|–|F_c|$ map contoured to 3.0σ and the

composite omit map contoured to 1.0σ as criteria. Final rounds of refinement included translation, libration, screw (TLS) parameterization with one TLS group per chain in the ASU.

For the substrate-bound structure, the one protomer in the ASU contains all residues in the sequence, 1–239 (out of 239) and 7 residues of N-terminal purification tag. Residues 176 – 183 were missing from the disordered region of helix α8, where no density was observed. The model additionally contained 99 water molecules and one D-malate ion.

For the substrate-bound structure with a partially-reconstituted cofactor, Chain A contains residues 1–238 (out of 239) and 1 residue of the N-terminal purification tag, and chain B contained residues 1–177 and 184–238 (out of 239) and two residues of the N-terminal purification tag. The missing residues (178–183) were from the disordered flexible region of helix α8. The structural model also contained per ASU 169 water molecules, one succinate ion, two D-malate ions, and one glycerol molecule. Following initial rounds of refinements, unaccounted for electron density in $2|F_o|–|F_c|$ maps calculated from native datasets was shown near the active site metal coordination spheres. Anomalous difference maps calculated from datasets collected at the Fe peak wavelength (1.7340 Å) showed consistent high σ electron density peaks: one at Fe in site 1 of chain A (~13 I/σ), one at Fe in site 1 of chain B (~12.5 I/σ), and one weaker peak at Fe in site 2 of chain B (~ 4.2 I/σ), indicating only partial occupancy. Fe ions were modeled in Coot into these electron density peaks. The structure contained three Fe ions (one in the active site of chain A and two in chain B). In chain B, the active site harbors two Fe ions for which the refined crystallographic B-factors are higher than that of Fe1 in chain A when all three Fe ions are set to full occupancy, suggesting partial occupancy of the two iron ions in chain B. To estimate iron occupancy, we set the B-factors of both iron in chain B to be the same as Fe1 in chain A, thereby calculating relative iron occupancy when Fe1 in chain A is assumed to be fully occupied. Refined occupancy shows that Fe1 in chain B and chain A have a similar high occupancy (~84%) and have the same ligands and coordination geometry. Fe2 has almost half of the occupancy (~43%). The identity of two irons in this chain was also confirmed by iron anomalous difference maps (see Figure 3).

For the substrate-bound structure with the fully-reconstituted cofactor, Chain A contains residues 1–238 (out of 239) and six residues of the N-terminal purification tag, and chain B contains residues 1–179 and 185–238 (out of 239) and three residues of the N-terminal purification tag. The missing residues (180–184) were from the disordered flexible region of

helix α8 where no density could be observed. The model additionally contained 261 water molecules. Unaccounted for, high σ electron density in $2|F_o|–|F_c|$ maps calculated from native datasets was observed in the cofactor site. Fe anomalous difference map shows consistent high-intensity peaks. Three Fe(II) ions were modeled in chain A and two were modeled in chain B. Iron occupancy was also estimated using the approach described above, where Fe1 in chain A is assumed to be full occupancy.

A summary of the data collection and refinement statistics for all reported structures can be found in Supplementary Table S1. All structural figures were generated using the PyMOL Molecular Graphics software package (Schrödinger, LLC). Crystallography and AlphaFold packages were compiled by SBGrid.[13]

Computational methods

All computations were performed with Gaussian 16.[14] Geometry optimizations and frequency calculations in water were conducted at the M06-2X/def2-TZVPP level of theory with the SMD solvation model.[15-19] For all stationary points, normal vibrational mode analysis was performed to confirm local minima. Conformation searches were carried out using Grimme's CREST conformer-rotamer ensemble sampling tool version 2.10.2 with xtb version 6.3.3.[20-22]

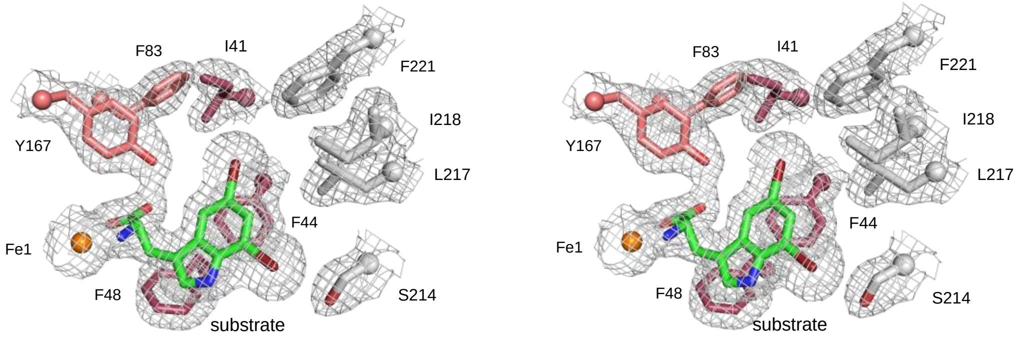

Supplementary Figure 1 Stereoview of substrate electron density. $2mF_o$-DF_c composite omit maps are contoured at 1.0σ The final refined model is superimposed. The image on the right is rotated 6 degrees from the left image.

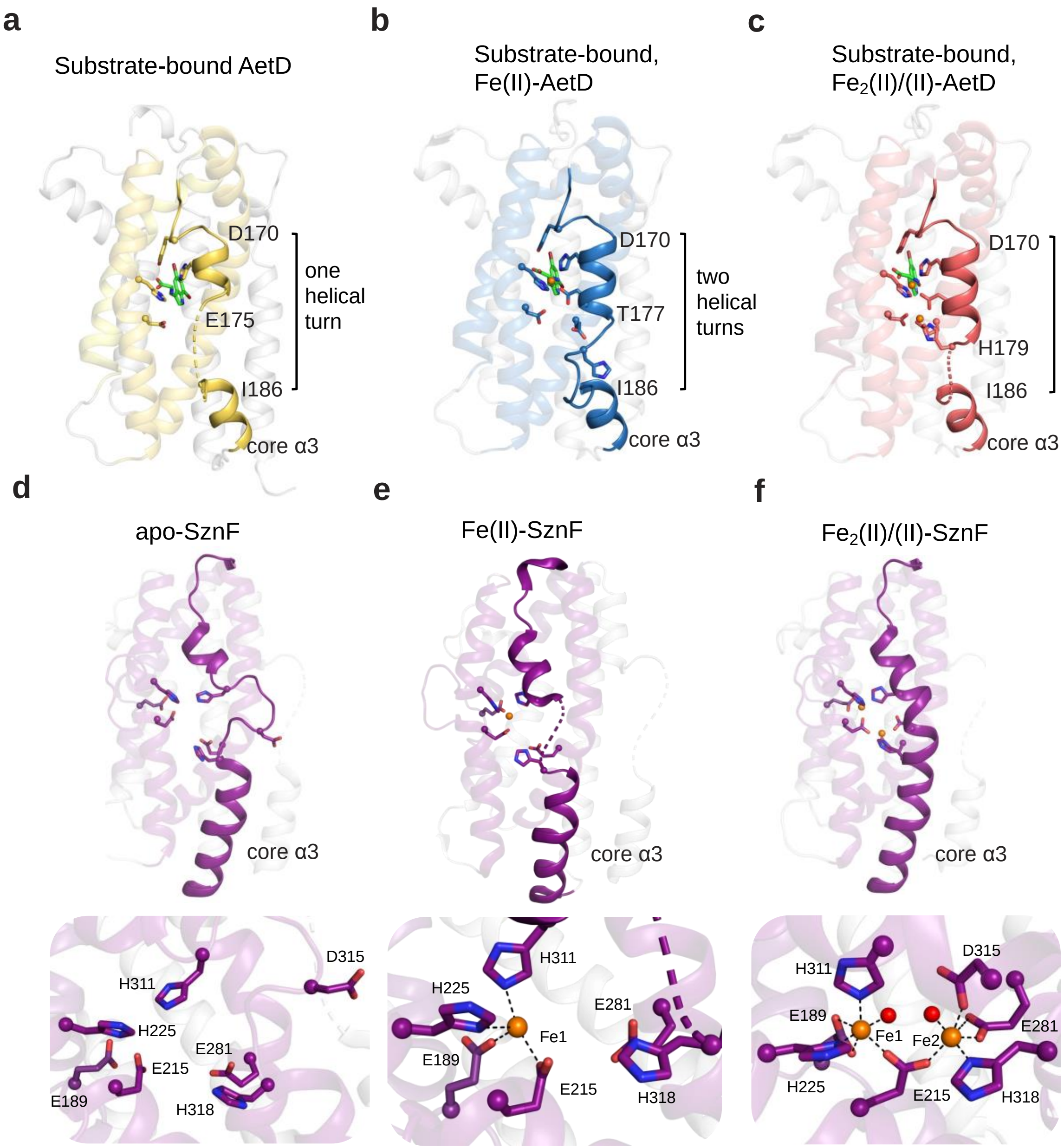

Supplementary Figure 2 Diiron cofactor assembly is conformationally gated by core helix α3. a-c, Core helix α3 becomes more ordered as the diiron cofactor is assembled. Segment containing residues D170 to H179 presents more ordered secondary structure as shown in increasing number of helical turns. **d-f,** Ordering of core helix α3 is also observed in substrate-independent SznF. When no iron or only one iron is bound at the cofactor, the helix is partially disordered; when both irons are bound, the entire helix becomes fully ordered. PDB IDs of different SznF structures are: **d,** 6XCV, **e,** 8E8W, and **f,** 6VZY.

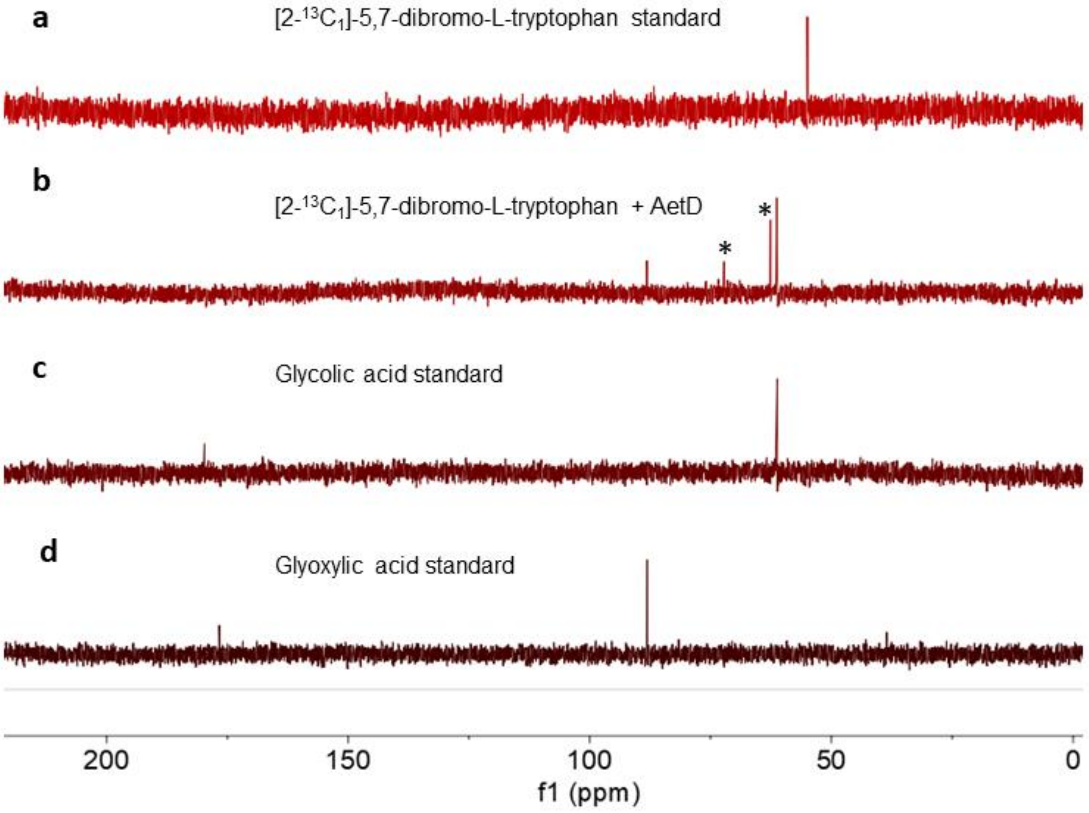

Su [illegible] **fragments.** Glycolic acid and glyoxylic acid were identified as the byproducts in the native and shunt pathways. **a,** [2-$^{13}C_1$]-5,7-dibromo-L-tryptophan standard. **b,** [2-$^{13}C_1$]-5,7-dibromo-L-tryptophan + AetD. **c,** Glycolic acid standard. **d,** Glyoxylic acid standard. When [2-$^{13}C_1$]-5,7-dibromo-L-tryptophan was incubated with AetD, two new NMR signals at 61 and 88 ppm appeared that match with glycolic acid (alcohol carbon) and glyoxylic acid (aldehyde carbon) standards, respectively. * Glycerol peak from the AetD protein stock.

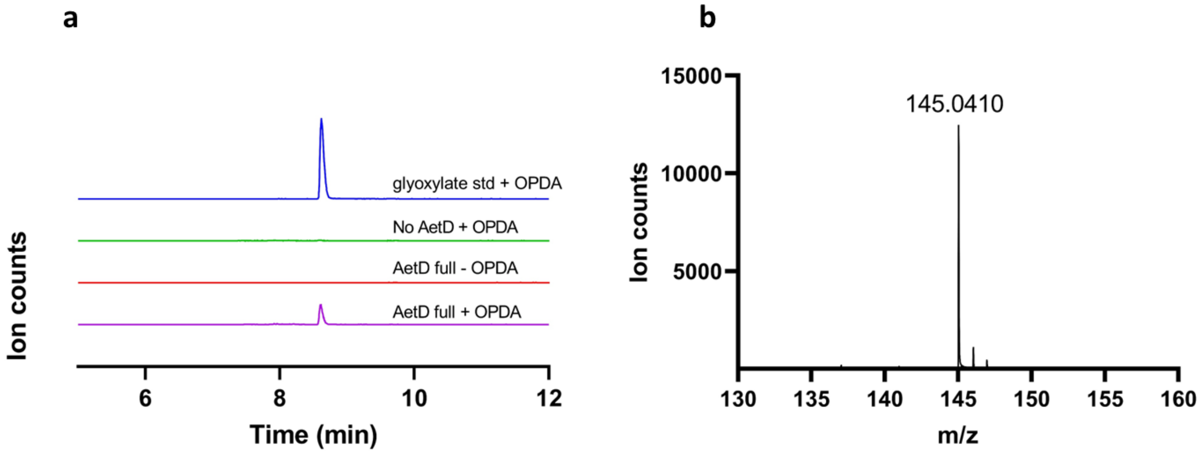

Supplementary Figure 4 Detection of glyoxylate using *o*-phenylenediamine derivatization. a, EICs at 145.04 corresponding to the *o*-phenylenediamine-glyoxylate $[M\text{-}H]^-$ adduct. The expected mass was only observed in the AetD full reaction in the presence of *o*-phenylenediamine. The mass cannot be detected in the absence of either AetD or *o*-phenylenediamine. **b,** The exact mass of *o*-phenylenediamine-glyoxylate adduct in negative ionization mode.

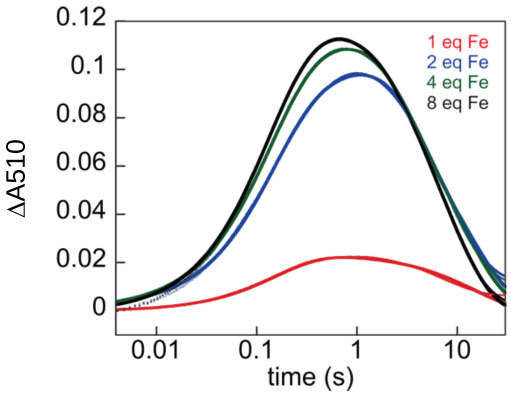

Supplementary Figure 5 Kinetic traces of the intermediate with absorption maxima λ= 510 nm. Kinetic traces of the intermediate with absorption maxima λ= 510 nm as a function of 1 molar eq Fe(II) (red trace), 2 molar eq Fe(II) (blue trace), 4 molar eq Fe(II) (green trace), and 8 molar eq Fe(II) (black trace). These traces were obtained after rapid mixing of an O_2-free solution of AetD (0.30 mM) and Fe(II) in the presence of 2 mM 5,7-dibromo-L-tryptophan with an equal volume of O_2-saturated buffer (1.8 mM).

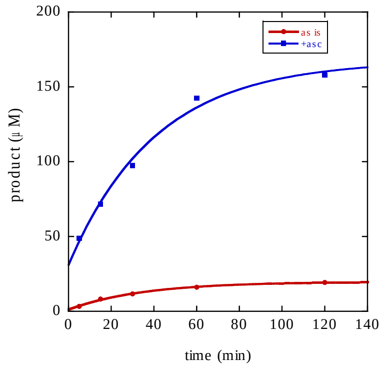

Supplementary Figure 6 Dependence of multiple turnover activity on the presence of a reducing agent (ascorbate). Experimental conditions: [AetD] = 20 μM, [Fe(II)] = 40 μM, [substrate] = 500 μM, [ascorbate] = 2 mM (when included), [O_2] = 1.6 mM.

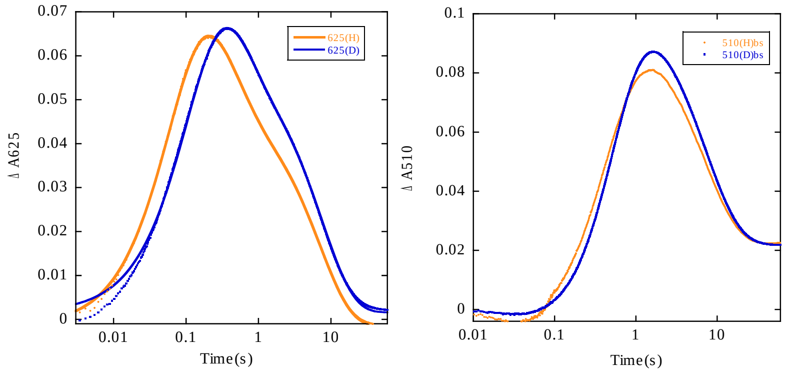

Supplementary Figure 7 Dependence of multiple turnover activity on the presence of a reducing agent. Kinetic traces of the intermediates with absorption maxima λ= 625 nm and λ= 510 nm after rapid mixing of an O_2-free solution of AetD (0.30 mM) and Fe(II) (0.6 mM) in the presence of 2 mM 5,7-dibromo-L-tryptophan (orange trace) or 2 mM [3-D_2]-5,7-dibromo-L-tryptophan (blue trace) with an equal volume of O_2-saturated buffer (1.8 mM). The estimated isotope effect KIE on the λ= 625 nm is 1.4 ± 0.3 and is lower than that previously reported for this intermediate for BesC,[23] whereas the λ= 510 nm species hardly exhibits any detectable KIE.

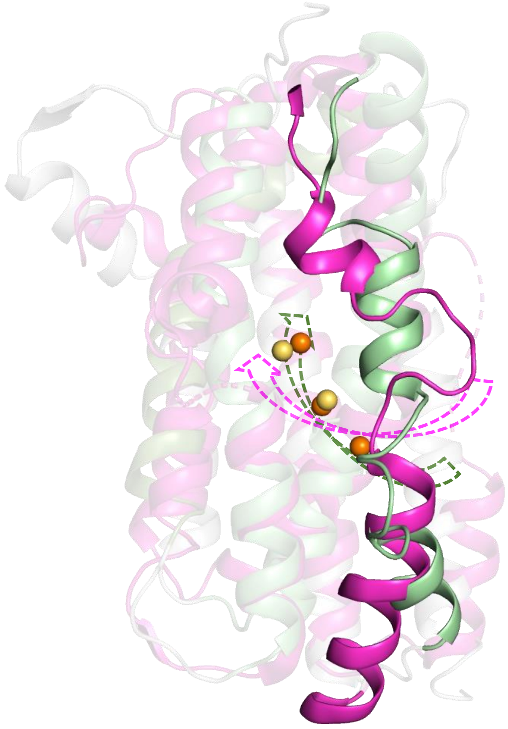

Supplementary Figure 8 Superimposition of AetD and SznF showing a possible route into the active site for Fe(II) ions when the core α3 helix is unwound. Structure of AetD with Fe(II) in three sites (this work) is in green ribbons and the structure of apo-SznF (PDB: 6M9R) is in purple ribbons. Core α3 helices are highlighted. Fe(II) in AetD is shown in orange spheres; Fe(II) in the structure of reconstituted SznF (PDB: 6VZY) is in yellow spheres. Dashed arrows indicate putative Fe access channels in AetD (green) and SznF (purple).

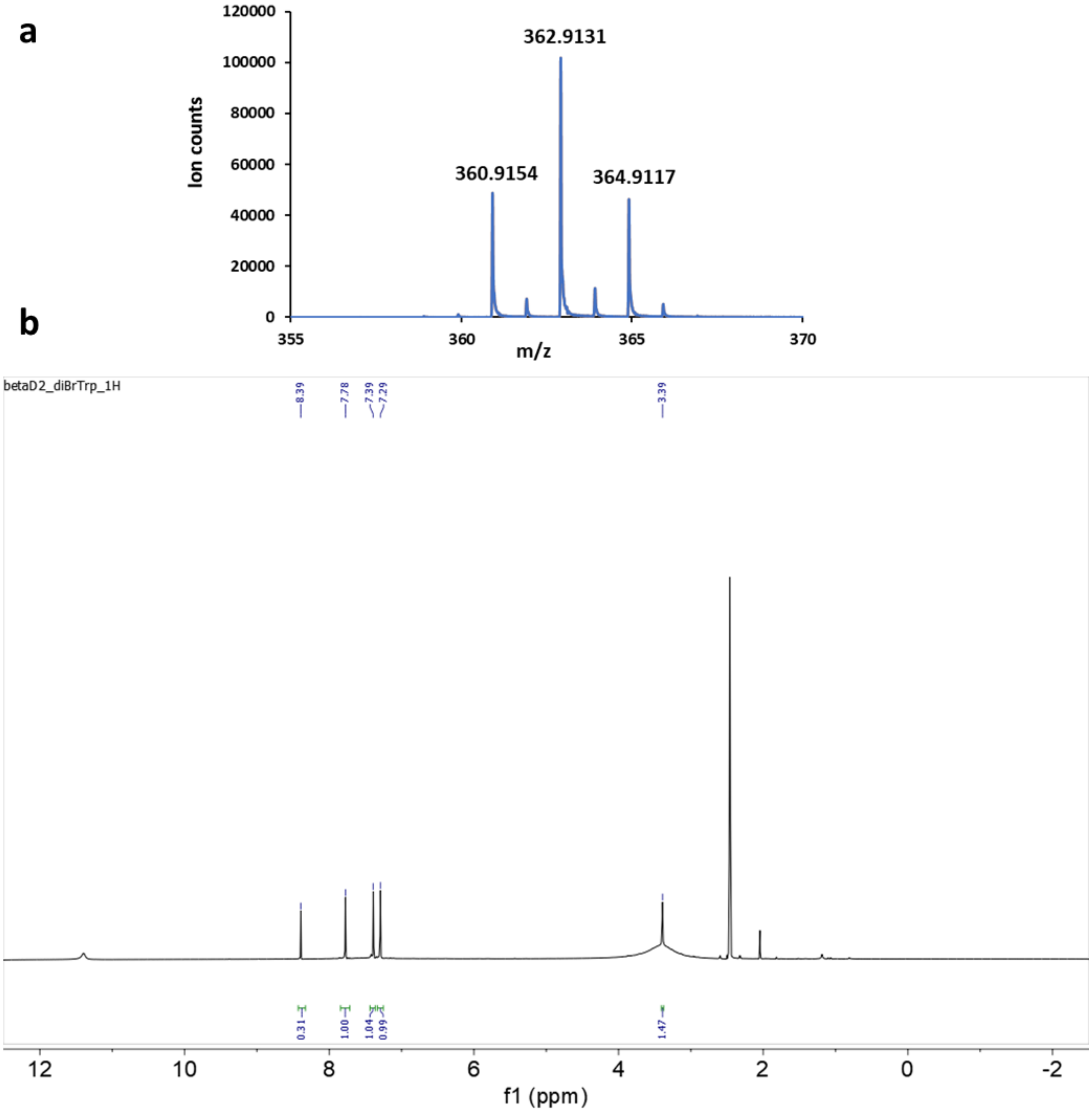

Supplementary Figure 9 Characterization of [3-D_2]-5,7-dibromo-L-tryptophan. a, High resolution mass spectrum of [3-D_2]-5,7-dibromo-L-tryptophan in negative ionization mode. *m/z: calcd for* $[M-H]^-$: 362.9141; found: 362.9131 **b,** ^{1}H-NMR spectrum of [3-D_2]-5,7-dibromo-L-tryptophan.

Supplementary Table 1 AetD data collection and refinement statistics

	Substrate-bound, Se-Met labeled	Substrate-bound	Substrate-bound and cofactor partially assembled		Substrate-bound and cofactor fully assembled	
**Data collection**						
Space group	$P\ 2_12_12_1$	$P\ 6_522$	$P\ 2_12_12_1$		$P\ 2_12_12_1$	
Cell dimensions						
a, *b*, *c* (Å)	65.7, 85.8, 101.7	101.5, 101.5, 129.1	65.9, 86.1, 101.8		65.9, 88.1, 102.8	
α, β, γ (°)	90, 90, 90	90, 90, 120	90, 90, 90		90, 90, 90	
			Native	*Fe peak**	*Native*	*Fe peak**
Beamline	SSRL 9-2*	APS 24-ID-C	SSRL 9-2	APS 24-ID-C	APS 24-ID-C	
Wavelength (Å)	0.97910	0.97918	0.95369	1.73404	0.97918	1.73404
Resolution (Å)	50.00-2.30 (2.44-2.30)	50.00-2.08 (2.21-2.08)	50.00-2.30 (2.44-2.30)	50.00-2.29 (2.42-2.29)	50.00-2.00 (2.13-2.00)	50.00-2.49 (2.55-2.49)
Observed Reflections	340402 (52130)	205075 (30288)	315639 (47689)	189131 (30769)	362648 (60171)	246120 (9565)
Unique Reflections	47930 (7654)	22444 (3470)	25947 (3958)	48395 (7823)	40402 (6430)	40417 (2907)
R_{sym} or R_{merge}	0.066 (0.304)	0.131 (0.752)	0.126 (0.626)	0.124 (0.660)	0.109 (0.543)	0.162 (0.554)
$I / \sigma I$	22.8 (6.1)	9.6 (1.6)	15.6 (4.0)	8.4 (2.2)	13.19 (4.2)	8.6 (3.1)
$CC_{1/2}$ (%)	0.999 (0.967)	0.995 (0.844)	0.998 (0.927)	0.993 (0.808)	0.997 (0.937)	0.987 (0.839)
Completeness (%)	96.7 (95.5)	92.4 (90.4)	97.8 (93.6)	95.0 (95.0)	98.7 (99.0)	99.4 (96.0)
Redundancy	7.1 (6.8)	9.1 (8.7)	12.2 (12.0)	3.9 (3.9)	9.0 (9.4)	6.1 (3.3)
**Refinement**						
Resolution (Å)		43.97-2.08	39.53-2.30		44.40-2.00	
No. reflections		22435	25940		40398	
No. reflections R_{free}		1122	1303		2022	
R_{work} / R_{free}		0.194/0.216	0.186/0.218		0.184/0.209	
No. atoms/molecules						
Protein chain per ASU		1	2		2	
Protein atom		1985	3936		3985	
Protein residue		238	473		480	
Iron ion		0	3		5	
Substrate molecule		1	2		2	
Succinate ion		0	1		0	
Malate ion		1	2		0	
Glycerol molecule		0	1		0	
Water molecule		99	169		261	
B-factors ($Å^2$)						
Protein Chains						
Chain A		46.25	34.47		32.03	
Chain B		-	36.82		32.43	
Iron ion		-	45.45		35.68	
Substrate molecule		44.43	28.48		26.75	
Succinate ion		-	55.04		-	
Malate ion		52.75	33.59		-	
Glycerol molecule		-	41.94		-	
Water molecule		51.17	35.94		36.77	
R.m.s deviations						
Bond lengths (Å)		0.002	0.002		0.004	
Bond angles (°)		0.59	0.58		0.73	
Ramachandran plot						
Favored (%)		99.15	98.72		98.95	
Allowed (%)		0.85	1.28		1.05	
Outliers (%)		0.00	0.00		0.00	

Rotamer outliers (%)	0.46	0.70	0.69
Clashscore	2.51	3.15	3.02
No. TLS groups	1	2	2

Values in parentheses are for highest-resolution shell. A single crystal was used to collect each dataset. *Statistics are for data processed anomalously (Friedel pairs scaled separately and unmerged).

Supplementary Table 2. Absolute calculation energies, enthalpies, and free energies at the SMD-M06-2X/def2-TZVPP level of theory.

Geometry	$E_{(solv,M06\text{-}2X)}$[1]	$H_{(solv,M06\text{-}2X)}$[2]	$G_{(solv,M06\text{-}2X)}$[3]
**7**	-5833.583725	0.216911	0.150239
**8**	-5603.259871	0.121341	0.070122
**11**	-304.327491	0.074071	0.038873
**CH3COOH**	-229.100810	0.067239	0.034634
**CH3COOOH**	-304.227608	0.071958	0.034301
**H2O**	-76.441453	0.025058	0.003633
**H3O**	-76.857458	0.038924	0.015989
**H-radical**	-0.495999	0.002360	-0.010654
**13**	-5832.935043	0.203364	0.138869
**14**	-5832.779343	0.206235	0.143031
**15**	-5832.795356	0.207455	0.145673
**16**	-5832.817825	0.207223	0.143583
**17(18)**	-5907.989346	0.211750	0.145625
**19**	-5831.570749	0.181734	0.116779

[1]The electronic energy calculated by SMD-M06-2X/def2-TZVPP in water solvent. [2]The thermal correction to enthalpy calculated by SMD-M06-2X/def2-TZVPP in water solvent. [3]The thermal correction to Gibbs free energy calculated by SMD-M06-2X/def2-TZVPP in water solvent.

References

1. Adak, S., Lukowski, A. L., Schäfer, R. J. B. & Moore, B. S. From Tryptophan to Toxin: Nature's Convergent Biosynthetic Strategy to Aetokthonotoxin. *J. Am. Chem. Soc.* **144**, 2861-2866, (2022).
2. Bhandari, D. M., Fedoseyenko, D. & Begley, T. P. Mechanistic Studies on Tryptophan Lyase (NosL): Identification of Cyanide as a Reaction Product. *J. Am. Chem. Soc.* **140**, 542-545, (2018).
3. Kabsch, W. xds. *Acta Crystallogr. D* **66**, 125-132 (2010).
4. Karplus, P. A. & Diederichs, K. Assessing and maximizing data quality in macromolecular crystallography. *Curr. Opin. Struc. Biol.* **34**, 60-68 (2015).
5. Grosse-Kunstleve, R. W. & Adams, P. D. Substructure search procedures for macromolecular structures. *Acta Crystallogr. D* **59**, 1966-1973 (2003).
6. McCoy, A. J., Storoni, L. C. & Read, R. J. Simple algorithm for a maximum-likelihood SAD function. *Acta Crystallogr. D* **60**, 1220-1228 (2004).
7. McCoy, A. J. *et al.* Phaser crystallographic software. *J. Appl. Crystallogr.* **40**, 658-674 (2007).
8. Terwilliger, T. C. Maximum-likelihood density modification. *Acta Crystallogr. D* **56**, 965-972 (2000).
9. Jumper, J. *et al.* Highly accurate protein structure prediction with AlphaFold. *Nature* **596**, 583-589 (2021).
10. Emsley, P., Lohkamp, B., Scott, W. G. & Cowtan, K. Features and development of Coot. *Acta Crystallogr. D* **66**, 486-501 (2010).
11. Moriarty, N. W., Grosse-Kunstleve, R. W. & Adams, P. D. electronic Ligand Builder and Optimization Workbench (eLBOW): a tool for ligand coordinate and restraint generation. *Acta Crystallogr. D* **65**, 1074-1080 (2009).
12. Terwilliger, T. C. *et al.* Iterative-build OMIT maps: map improvement by iterative model building and refinement without model bias. *Acta Crystallogr. D* **64**, 515-524 (2008).
13. Morin, A. *et al.* Collaboration gets the most out of software. *elife* **2**, e01456 (2013).
14. Frisch, M. J. et al. Gaussian 16 Revision A.03, Gaussian, Inc.,Wallingford, CT (2016).
15. Zhao, Y., & Truhlar, D. G. Density Functionals with Broad Applicability in Chemistry. *Acc. Chem. Res.* **41**, 157–167 (2008).
16. Zhao, Y., & Truhlar, D. G. The M06 Suite of Density Functionals for Main Group Thermochemistry, Thermochemical Kinetics, Noncovalent Interactions, Excited States, and Transition Elements: Two New Functionals and Systematic Testing of Four M06-Class Functionals and 12 Other Functionals. *Theor. Chem. Acc.* **120**, 215–241 (2008).
17. Marenich, A. V., Cramer, C. J., & Truhlar, D. G. Universal Solvation Model Based on Solute Electron Density and on a Continuum Model of the Solvent Defined by the Bulk Dielectric Constant and Atomic Surface Tensions. *J. Phys. Chem. B* **113**, 6378–6396 (2009).
18. Schäfer, A., Huber, C., & Ahlrichs, R. Fully Optimized Contracted Gaussian Basis Sets of Triple Zeta Valence Quality for Atoms Li to Kr. *J. Chem. Phys.* **100**, 5829–5835 (1994).
19. Weigend, F., & Ahlrichs, R. Balanced Basis Sets of Split Valence, Triple Zeta Valence and Quadruple Zeta Valence Quality for H to Rn: Design and Assessment of Accuracy. *Phys. Chem. Chem. Phys.* **7**, 3297–3305 (2005).
20. Grimme, S. Exploration of chemical compound, conformer, and reaction space with metadynamics simulations based on tight-binding quantum chemical calculations. *J. Chem. Theory Comput.* **15**, 2847–2862 (2019).
21. Grimme, S., Bannwarth, C., & Shushkov, P. A robust and accurate tight-binding quantum chemical method for structures, vibrational frequencies, and noncovalent

interactions of large molecular systems parametrized for all spd-block elements (Z = 1–86). *J. Chem. Theory Comput*. **13***,* 1989–2009 (2017).

22. Bannwarth, C., Ehlert, S., & Grimme, S. GFN2-xTB–An accurate and broadly parametrized self-consistent tight-binding quantum chemical method with multipole electrostatics and densitydependent dispersion contributions. *J. Chem. Theory Comput*. **15**, 1652–1671 (2019).
23. McBride, M. J. *et al.* Substrate-Triggered µ-Peroxodiiron(III) Intermediate in the 4-Chloro-l-Lysine-Fragmenting Heme-Oxygenase-like Diiron Oxidase (HDO) BesC: Substrate Dissociation from, and C4 Targeting by, the Intermediate. *Biochemistry* **61**, 689-702, (2022).
