# Supplementary material for: A single diiron enzyme catalyses the oxidative rearrangement of tryptophan to indole nitrile
Source: Nat Chem. 2024 Sep 16;16(12):1989–98. doi: 10.1038/s41557-024-01603-z (PMC11611611; doi:10.1038/s41557-024-01603-z)
Supplement: Supplementary file 2 — Reporting Summary [file 41557_2024_1603_MOESM2_ESM.pdf]

nature portfolio

Corresponding author(s): Bradley S. Moore

Last updated by author(s): Jun 26, 2024

Reporting Summary

Nature Portfolio wishes to improve the reproducibility of the work that we publish. This form provides structure for consistency and transparency in reporting. For further information on Nature Portfolio policies, see our Editorial Policies and the Editorial Policy Checklist.

Statistics

For all statistical analyses, confirm that the following items are present in the figure legend, table legend, main text, or Methods section.

n/a	Confirmed	
☐	☒	The exact sample size (n) for each experimental group/condition, given as a discrete number and unit of measurement
☐	☒	A statement on whether measurements were taken from distinct samples or whether the same sample was measured repeatedly
☒	☐	The statistical test(s) used AND whether they are one- or two-sided<br>*Only common tests should be described solely by name; describe more complex techniques in the Methods section.*
☒	☐	A description of all covariates tested
☒	☐	A description of any assumptions or corrections, such as tests of normality and adjustment for multiple comparisons
☒	☐	A full description of the statistical parameters including central tendency (e.g. means) or other basic estimates (e.g. regression coefficient) AND variation (e.g. standard deviation) or associated estimates of uncertainty (e.g. confidence intervals)
☒	☐	For null hypothesis testing, the test statistic (e.g. F, t, r) with confidence intervals, effect sizes, degrees of freedom and P value noted<br>*Give P values as exact values whenever suitable.*
☒	☐	For Bayesian analysis, information on the choice of priors and Markov chain Monte Carlo settings
☒	☐	For hierarchical and complex designs, identification of the appropriate level for tests and full reporting of outcomes
☒	☐	Estimates of effect sizes (e.g. Cohen's d, Pearson's r), indicating how they were calculated

Our web collection on statistics for biologists contains articles on many of the points above.

Software and code

Policy information about availability of computer code

Data collection	Crystallography: X-ray diffraction data were collected at Advanced Photon Source (APS) beamline 24-ID-C using NE-CAT remote access program, and at Stanford Synchrotron Light Source (SSRL) beamline 9-2 using NoMachine remote client program.
Data analysis	Spectroscopic analysis: MestreNova® 14.21-27684, 2021 for NMR analysis, MassHunter Workstation Software version B.05.01.for LCMS analysis. Crystallography: X-ray Detector Software (XDS) package (version 20230630) was used for indexing, integrating, and scaling. The following programs in the Phenix package (version 1.20.1-4487) were used for crystal structure determination: Hybrid Substructure Search (HySS) for finding Se positions, Phaser-EP for experimental Se-Met SAD phasing, phenix.RESOLVE for density modifications, and phenix.refine for refinements. Coot (version 0.9.8.8) was used for structural model building. XDS and Phenix softwares are compiled by SBGrid. PyMOL (version 2.4.0) was used to produce structural figures.

For manuscripts utilizing custom algorithms or software that are central to the research but not yet described in published literature, software must be made available to editors and reviewers. We strongly encourage code deposition in a community repository (e.g. GitHub). See the Nature Portfolio guidelines for submitting code & software for further information.

Data

Policy information about availability of data

All manuscripts must include a data availability statement. This statement should provide the following information, where applicable:

- Accession codes, unique identifiers, or web links for publicly available datasets
- A description of any restrictions on data availability
- For clinical datasets or third party data, please ensure that the statement adheres to our policy

NCBI accession number of AetD sequence used in this study is QNL15174. Atomic coordinates and structure factors for the crystal structures reported in this work have been deposited to the Protein Data Bank (PDB) under accession numbers 8TWN (substrate-bound AetD), 8TWT (substrate-bound AetD with diiron cofactor partially assembled), and 8TWW (substrate-bound AetD with diiron cofactor fully assembled). Additionally, the following PDB structures were used for AetD structural comparison in this paper: 1WOW, 1RCW, 6P5Q, 7TWA, 6VZY, 6M9R, and 6M9S. Source data are provided with this paper. Other relevant data supporting the findings of this study are available in this published article or its Supplementary files.

Human research participants

Policy information about studies involving human research participants and Sex and Gender in Research.

Reporting on sex and gender	n/a
Population characteristics	n/a
Recruitment	n/a
Ethics oversight	n/a

Note that full information on the approval of the study protocol must also be provided in the manuscript.

Field-specific reporting

Please select the one below that is the best fit for your research. If you are not sure, read the appropriate sections before making your selection.

☒ Life sciences ☐ Behavioural & social sciences ☐ Ecological, evolutionary & environmental sciences

For a reference copy of the document with all sections, see nature.com/documents/nr-reporting-summary-flat.pdf

Life sciences study design

All studies must disclose on these points even when the disclosure is negative.

Sample size	n/a
Data exclusions	No data were excluded
Replication	All in vitro enzyme assay were performed in at least 2 independent experiments (N = 2) with replicates (n=2). All attempts at replication were successful.
Randomization	Randomization was not relevant as no live animal or human subjects were involved.
Blinding	Blinding was not relevant as the data was collected and analyzed using computer software.

Reporting for specific materials, systems and methods

We require information from authors about some types of materials, experimental systems and methods used in many studies. Here, indicate whether each material, system or method listed is relevant to your study. If you are not sure if a list item applies to your research, read the appropriate section before selecting a response.

Materials & experimental systems

n/a	Involved in the study
☒	☐ Antibodies
☒	☐ Eukaryotic cell lines
☒	☐ Palaeontology and archaeology
☒	☐ Animals and other organisms
☒	☐ Clinical data
☒	☐ Dual use research of concern

Methods

n/a	Involved in the study
☒	☐ ChIP-seq
☒	☐ Flow cytometry
☒	☐ MRI-based neuroimaging
